# The association between human papillomavirus and bladder cancer: Evidence from meta‐analysis and two‐sample mendelian randomization

**DOI:** 10.1002/jmv.28208

**Published:** 2022-10-25

**Authors:** Jian‐Xuan Sun, Jin‐Zhou Xu, Chen‐Qian Liu, Ye An, Meng‐Yao Xu, Xing‐Yu Zhong, Na Zeng, Si‐Yang Ma, Hao‐Dong He, Jia Hu, Zheng Liu, Shao‐Gang Wang, Qi‐Dong Xia

**Affiliations:** ^1^ Department and Institute of Urology, Tongji Hospital, Tongji Medical College Huazhong University of Science and Technology Wuhan China

**Keywords:** bladder cancer, HPV prevalence, human papillomavirus, mendelian randomization, meta‐analysis

## Abstract

**Introduction:**

Bladder cancer (BCa) is the 10th most common type of cancer worldwide, and human papillomavirus (HPV) is the most common sexually transmitted infection. However, the relationship between HPV infection and the risk of BCa is still controversial and inconclusive.

**Methods:**

This systematic review and meta‐analysis were conducted following the PRISMA 2020 reporting guideline. This study searched four bibliographic databases with no language limitation. The databases included PubMed (Medline), EMBASE, Cochrane Library, and Web of Science. Studies evaluating the interaction between HPV infection and the risk of BCa from inception through May 21, 2022, were identified and used in this study. This study estimated the overall and type‐specific HPV prevalence and 95% confidence intervals (95% CI) using Random Effects models and Fixed Effects models. In addition, this study also calculated the pooled odds ratio and pooled risk ratio with 95% CI to assess the effect of HPV infection on the risk and prognosis of bladder cancer. Two‐sample mendelian randomization (MR) study using genetic variants associated with HPV E7 protein as instrumental variables were also conducted.

**Results:**

This study retrieved 80 articles from the four bibliographic databases. Of the total, 27 were case–control studies, and 53 were cross‐sectional studies. The results showed that the prevalence of HPV was 16% (95% CI: 11%–21%) among the BCa patients, most of which were HPV‐16 (5.99% [95% CI: 3.03%–9.69%]) and HPV‐18 (3.68% [95% CI: 1.72%–6.16%]) subtypes. However, the study found that the prevalence varied by region, detection method, BCa histological type, and sample source. A significantly increased risk of BCa was shown for the positivity of overall HPV (odds ratio [OR], 3.35 [95% CI: 1.75–6.43]), which was also influenced by study region, detection method, histological type, and sample source. In addition, the study found that HPV infection was significantly associated with the progression of BCa (RR, 1.73 [95% CI: 1.39–2.15]). The two‐sample MR analysis found that both HPV 16 and 18 E7 protein exposure increased the risk of BCa (HPV 16 E7 protein: IVW OR per unit increase in protein level = 1.0004 [95% CI: 1.0002–1.0006]; *p* = 0.0011; HPV 18 E7 protein: IVW OR per unit increase in protein level = 1.0003 [95% CI: 1.0001–1.0005]; *p* = 0.0089).

**Conclusion:**

In conclusion, HPV may play a role in bladder carcinogenesis and contribute to a worse prognosis for patients with BCa. Therefore, it is necessary for people, especially men, to get vaccinated for HPV vaccination to prevent bladder cancer.

## 1 INTRODUCTION

Bladder cancer (BCa) is the 10th most common cancer worldwide, accounting for approximately 550 000 new cases annually^1^ and an estimated 17 980 deaths in the United States in 2020.^2^ Many studies have found that BCa is the most costly cancer resulting in a heavy economic burden on society and individuals. For instance, it has been estimated that BCa treatment costs the US government about $4 billion annually.^1^ Bladder cancer can be divided into non‐muscle invasive bladder cancer (NMIBC) and muscleinvasive bladder cancer (MIBC). This categorization depends on whether the tumor has invaded the muscular layer of the bladder. Studies have found that nearly 75% of BCa cases are NMIBC and the rest are MIBC.^3^ Histologically, BCa consists of urothelial carcinoma (UC), squamous cell carcinoma (SCC), and adenocarcinoma, among which UC accounts for 94% of all cases.^4^ On the other hand, studies have also found that tobacco smoking, occupational exposure to several chemical compounds such as aromatic amines and arsenic,^5^, ^6^ and genetic factors may contribute to a higher incidence of BCa.^3^

Human papillomavirus (HPV), a family member of papillomaviruses, is a DNA virus that infects the cutaneous or mucosal epithelium. Studies have found that HPV is the most common sexually transmitted infection among humans.^7^ For instance, it has been reported that about 45.2% of men between 18 and 59 years in the United States have an HPV infection.^8^ In contrast, about 80% of productive women have a lifetime risk of developing an HPV infection.^9^ More than 100 HPV genotypes have been identified so far and can be divided into low‐risk and high‐risk groups according to their oncogenic potential.^9^ Low‐risk HPV types such as HPV‐6 and 11 cause recurrent respiratory papillomatosis (RRP) and anogenital warts that rarely develop into cancer. On the other hand, high‐risk HPV types, such as HPV‐16, 18, 31, and 33, cause about 10% of cancers worldwide, including more than 90% of cervical cancers, most anal cancers, and a part of vulvar, vaginal, and penile cancers.^4^, ^10^

The urinary tract is anatomically close to the genital tract. As a result, HPV has a high probability of infecting the urinary epithelium of the bladder.^11^, ^12^ Previous studies have reported that primary penile cancer was positively associated with high‐risk HPV infection.^13^ Many studies have demonstrated how HPV infection influences the development and prognosis of BCa. These studies identified the chronic inflammation caused by HPV infection as a significant risk factor for tumorigenesis. The studies also reported the reactive oxygen species (ROS) and reactive nitrogen species (RNS) generated in epithelial cells cause oxidative and nitrative DNA damage, mutations, and carcinogenesis during cell inflammation.^14^ On the other hand, it has been reported that E6 and E7 viral oncoproteins play important roles in tumorigenesis. The HPV genome can integrate into the host genome and increase the expression of E6 and E7 proteins. This will stimulate cell growth, inhibit differentiation, and induce chromosomal instability, leading to tumorigenesis.^15^ Shaker et al. found that HPV 6/11 and 16/18 infections were higher in bilharzial bladder carcinoma than in chronic cystitis. Therefore, the authors concluded that HPV 6/11 and 16/18 contribute to bilharzial bladder tumorigenesis.^16^ A study conducted on a Chinese population, reported that HPV 18, 33, 16, and 39 contribute to the carcinogenesis of the bladder in both genders.^17^ However, some studies found different results. For example, a multi‐institutional study found that high‐risk HPV infection is not associated with an increased risk of the SCC of the bladder.^18^ Although many studies have been conducted, scientists are yet to conclusively explain how HPV increases the risk of developing BCa. A meta‐analysis including 52 studies published in 2011 reported that the prevalence of HPV in patients with BCa was 16.88% (95% confidence interval [CI], 15.53%–18.31%). The study concluded that HPV increases the incidence of BCa (odds ratio [OR], 2.84 [95% CI: 1.39–5.80]).^19^ Many studies have been published since then and their results have been found to be controversial and inconclusive. The results are controversial and inconclusive because they are primarily affected by HPV genotypes, regions, and detection methods.

Mendelian randomization (MR) is a method that uses genetic variants as instrumental variables of the exposure to evaluate the causal effects of the exposure on an outcome to exclude the influence of the confounding factors inherent in observational studies.^20^ On the other hand, the two‐sample MR analysis uses summary statistics of genome‐wide association studies (GWASs) instead of analyzing individual‐level data. Therefore, to fill this knowledge gap on the association between HPV infection and BCa, this systematic review and meta‐analysis and two‐sample MR analyses were conducted to reevaluate the relationship between HPV infection and the risk of BCa.

## 2 MATERIALS AND METHODS

### 2.1 Selection criteria

The inclusion and exclusion criteria were as follows: (1) The exposure of interest was HPV infection in bladder tissues; (2) The outcome of interest was bladder cancer; (3) This study only included the latest patient datasets reported in different articles; (4) Immunosuppressed patients such as those accepting organ transplant or with immunodeficiency were excluded; (5) Patients with secondary malignant tumors of the bladder and cervical lesions simultaneously were excluded; (6) Studies using cell or animal models and case reports were excluded; (7) Studies without full text or lack of valuable data were excluded. The search was not limited to any language or publication status.

### 2.2 Search strategy

**Figure 1:**
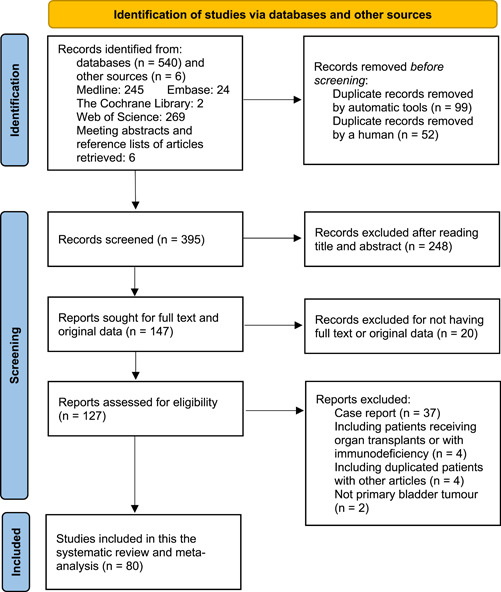
PRISMA (Preferred Reporting Items for Systematic Reviews and Meta‐Analyses) flowchart for study selection for the systematic review on HPV infection and the risk of bladder cancer. HPV, human papillomavirus.

This study was conducted according to the Preferred Reporting Items for Systematic Reviews and Meta‐analyses (PRISMA) 2020 reporting guideline.^21^ Four bibliographic databases, PubMed (Medline), EMBASE, Cochrane Library, and Web of Science, were searched to retrieve studies evaluating the interaction between HPV infection and the risk of bladder cancer from inception through May 21, 2022. The study also searched Google Scholar to retrieve gray literature, such as conference abstracts. The reference lists of articles, reviews, and meta‐analyses retrieved from these databases were manually screened to identify potentially relevant studies. The databases were searched using keywords such as “Papillomavirus Infections,” “HPV Infection,” “Bladder Cancer,” and “Urinary Bladder Neoplasms.” Supporting Information: Table S1 contains the detailed search strategy for each database, including the keywords used and the number of retrieved citations per string. Two reviewers, S. J. X. and X. Q. D., searched abstracts during the screening procedure and selected them according to the search criteria. Discrepancies about the inclusion or exclusion were resolved by consensus of the third author (L. C. Q.), and the Endnote application (version X9) was used to remove the duplicates and apply the inclusion criteria. A PRISMA flow chart in Figure 1 was used to depict the literature search procedure. This systematic review and meta‐analysis study was registered in PROSPERO (CRD42022330965).

### 2.3 Data extraction

This study used a designed data extraction sheet to extract information from the included studies. The data extraction sheet consisted of bibliographic information and background information. Bibliographic information had author's name, year of publication, journal name, and title. Background information included the country where the study was carried out, the inclusion and exclusion criteria for patients, age, percentage of male patients, and the smoking rate of the patients. In addition, background information had sample source, detection method, types of HPV detected, histological types of bladder cancer, the prevalence of HPV (overall and type‐specific) in bladder tissues, matching criteria if the study design was case–control and follow‐up duration, and the outcome of patients. Moreover, if the prevalence of HPV was detected using two or more methods, such as polymerase chain reaction (PCR) and in situ hybridization (ISH), the results of PCR were used for further analyses. The detailed characteristics of the articles included in this study are shown in Table 1. The outcomes of interest were the prevalence of HPV, the risk of bladder cancer, and the prognosis of bladder cancer patients.

**Table 1:** Characteristics of included studies in this systematic review and meta‐analysis

Study (year)	Country	Study type	Patient charactersitics	Age (years), median (range) or mean (SD)	Percentage of male	Smoking rate	Sample source	Detection Method	HPV Types detected	Histological type	Match
Yildizhan et al. (2021)^22^	Turkey	Case–control	Patients operated in Ankara City Hospital between 2009 and 2014	68.5 (24–89)	81.40%	100%	Biopsy, fixed tissue	PCR	18 high‐risk HPV types (16, 18, 26, 31, 33, 35, 39, 45, 51, 52, 53, 56, 58, 59, 66, 68, 69 and 73) together with the possible high‐risk type 34, and 13 low‐risk group HPV types (6, 11, 32, 40, 42, 43, 44, 54, 70, 72, 81, 84, and 87)	TUBC	patients with benign bladder pathologies (inflammation, inverted papilloma, papilloma, and cystitis)
Yan et al. (2021)^17^	China	Cross‐sectional study	Patients with bladder cancer pathological diagnosis admitted in the General Hospital of the People's Liberation Army (Beijing, China) between 2015 and 2019	66.64 ± 10.06	83.60%	47.62%	Biopsy, frozen tissue	PCR, qPCR	17 HPV genomes (HPV6, 11, 16, 18, 31, 33, 35, 39, 45, 52, 56, 58, 59, 66, 68, 69, and 82)	TUBC	NA
Sarier et al. (2021)^23^	Turkey	Cross‐sectional study	Patients who were diagnosed as having a primary or recurrent bladder tumor by ultrasound and/or cystoscopic examination in the urology outpatient clinic and underwent transurethral resection of bladder tumor (TUR‐BT) between January 1 and November 30, 2018	61.4 ± 13.9	84.21%	78.90%	First morning urine and urethral swab sample	PCR	low‐risk (types 6, 11, 44) and high‐risk (types 16, 18, 26, 31, 33, 35, 39, 45, 51, 52, 53, 56, 58, 59, 66, 68, 73, 82) HPV	stage pTa and pT1 UCB	NA
Musangile et al. (2021)^24^	Japan	Cross‐sectional study	Patients with UC in Wakayama Medical University and Kyorin University between 2013 and 2019; Cases of pure squamous cell carcinoma, adenocarcinoma, or neuroendocrine tumors of the bladder were excluded	74.8 ± 9.1	79.82%	58%	Biopsy, fixed tissue	RISH; IHC; PCR	RISH can recognize 18 HR (high‐ risk) HPV genotypes, which included the following: 16, 18, 26, 31, 33, 35, 39, 45, 51, 52, 53, 56, 58, 59, 66, 68, 73, and 82; IHC was used to detect the following 11 HPV genotypes: 6, 11, 16, 18, 31, 33, 42, 51, 52, 56, and 58; PCR were designed for 39 common HPV types	UC	NA
Sarier et al. (2020)^25^	Turkey	Case–control	Patients who underwent transurethral resection of bladder tumour at Medical Park Hospital between January and December 2018 and were diagnosed with UCB with muscle invasion according to the pathology report; those diagnosed with tumors other than UCB and who had previously undergone radical cystectomy were excluded	63.2 ± 12.6	84.06%	65.20%	Urethral swab samples and first morning void urine (15 ml) samples	PCR	low‐risk HPVs (Types 6, 11, and 44) and high‐risk HPVs (Types 16, 18, 26, 31, 33, 35, 39, 45, 51, 52, 53, 56, 58, 59, 66, 68, 73, and 82)	UCB	NA
Moghadam et al. (2020)^26^	Iran	Cross‐sectional study	Patients with histopathologically confirmed TCC of the urinary bladder	62.98 ± 10.26	80.19%	91.67%	Biopsy, fixed tissue	PCR; ISH	all recognized HPV types	TCC	NA
Gordetsky et al. (2020)^18^	USA	Cross‐sectional study	Patients at seven academic institutions with SCC of the bladder diagnosed between 1980 and 2019 that had undergone surgical resection by means of partial or radical cystectomy	66 ± 13.1	56.52%	64.25%	Biopsy, fixed tissue	ISH	genotypes 16, 18, 31, 33, 35, 45, 52, and 58	SCC	NA
Collins et al. (2020)^27^	USA	Cross‐sectional study	Patients with squamous cell carcinoma of the urinary bladder at the University of Texas MD Anderson Cancer from January 2009 to July 2019	64 (59–91)	48.48%	NA	Biopsy, fixed tissue	IHC for p16; RISH	HPV genotypes 16, 18, 31, 33, 35, 45, 42, and 58	SCC	NA
Javanmard et al. (2019)^28^	Iran	Cross‐sectional study	Patients who underwent transurethral resection of bladder tumor (TURBT) between January 2014 to May 2016 with the diagnosis of bladder TCC	61.6 ± 10	87.27%	61.82%	Biopsy, fixed tissue and urine samples	PCR	general HPV and HPV16 and 18 subtypes	TCC	NA
Llewellyn et al. (2018)^29^	UK	Cross‐sectional study	Patient in the Bladder Cancer Prognosis Programme (BCPP)	NA	78.52%	NA	Biopsy, frozen tissue	qPCR	HPV16 and HPV18	UBC	NA
Jørgensen et al. (2018)^30^	Denmark	Cross‐sectional study	Patients with bladder cancer from local databases of four Danish Departments of Pathology (Herlev University Hospital, Rigshospitalet, Odense University Hospital, and Aarhus University Hospital)	60.6 (56.5–64.7)	55.00%	90.91%	Biopsy, fixed tissue	PCR; IHC	Low risk (LR): HPV‐6, ‐11, ‐40, ‐42, ‐43, ‐44, ‐54, ‐61, ‐81; probable high risk (pHR): HPV‐26, ‐53, ‐66, ‐70, ‐73, ‐82; HR: HPV‐16, ‐18, ‐31, ‐33, ‐35, ‐39, ‐45, ‐51, ‐52, ‐56, ‐58, ‐59, ‐68; unknown risk: HPV‐62, ‐67, ‐83/‐52, ‐89	SCC, UC with squamous differentiation, UC	NA
Abdollahzadeh et al. (2017)^31^	Iran	Cross‐sectional study	Patients with TCC in Imam Reza Hospital medical center from 2008 to 2011	NA	86.57%	NA	Biopsy, fixed tissue	IHC	NA	TCC	NA
Golovina et al. (2016)^32^	Russia	Cross‐sectional study	Patients after transurethral resection of the bladder performed in Department of Urology in N. N. Blokhin Russian Cancer Research Center	61.7 (46–79)	83.17%	NA	Specimens after transurethral resection	PCR	HPV16	TCC	NA
Schmid et al. (2015)^33^	Germany	Case–control	Patients with urothelial carcinoma of the bladder undergoing cystectomy or transurethral resection of the bladder (TURB) at the Department of Urology, University Hospital rechts der Isar of the Technische Universität München	73 (45–94)	80.73%	NA	Biopsy, fixed tissue	PCR	high risk HPV genotypes (16, 18, 31, 33, 35, 39, 45, 51, 52, 56, 58, 59, 66, 68) and 35 low risk HPV genotypes (6, 7, 11, 13, 30, 32, 34, 40, 42, 43, 44, 53, 54, 55, 57, 61, 62, 67, 69, 70, 72, 73, 74, 81, 82, 83, 84, 85, 86, 87, 90, 91, 97, 102, 106)	UCB	NA
Pichler et al. (2015)^34^	Austria	Cross‐sectional study	Patients underwent transurethral resection of the bladder (TURB)due to primary non‐muscle invasive bladder cancer (NMIBC) at the Urology Department of Innsbruck	72 (24 – 93)	80.11%	NA	Biopsy, fixed tissue	PCR	40 genotypes: 6, 11, 16, 18, 26, 31, 33, 35, 39, 40, 42, 43, 44, 45, 51, 52, 53, 54, 55, 56, 58, 59, 61, 62, 64, 66, 67, 68a/b, 69, 70, 71, 72, 73, 81, 82, 83, 84, 87, 89, and 90	UC	NA
Piaton et al. (2014)^35^	France	Cross‐sectional study	patients being referred for cystoscopy at 2 Departments of Urology (Centre Hospitalier Lyon Sud, Pierre Benite, and Hopital Edouard Herriot, Lyon, France)	70 ± 2.7	81.71%	NA	Urine samples and tissue sample	PCR; ISH	35 HPVs: 20 high‐risk types (HPV‐16, ‐18, ‐26, ‐31, ‐33, ‐35, ‐39, ‐45, ‐51, ‐52, ‐53, ‐56, ‐58, ‐59, ‐66, ‐68, ‐70, ‐73, ‐82, and ‐85) and 15 low‐risk types (HPV‐6, ‐11, ‐40, ‐42, ‐43, ‐44, ‐54, ‐61, ‐62, ‐71, ‐72, ‐81, ‐83, ‐84, and ‐89)	UC	Cystoscopy and cytology both negative, without histology (20 cases), or negative cytology confirmed by negative histology control (chronic cystitis, 3 cases)
Kim et al. (2014)^36^	Korea	Case–control	Patients in Center for Prostate Cancer, National Cancer Center from July 2001 to March 2011	71.2 ± 7.7	82.86%	68.97%	Biopsy, fixed tissue	PCR; IHC	high‐risk HPV types (HPV‐16, 18, 31, 33, 35, 39, 45, 51, 52, 53, 56, 58, 59, 66, and 68) and low‐risk HPV types (HPV‐6, 11, 34, 40, 42, 43, 44, 54, and 70)	UC/SCC	patients with squamous metaplasia of the bladder
Alexander et al. (2014)^37^	USA	Cross‐sectional study	Patients from Indiana University, Cordoba University, and Polytechnic University of the March Regions	61 (32 – 87)	60%	NA	Biopsy, fixed tissue	IHC; ISH	HPV subtypes 6, 11, 16, 18, 31, 33, 42, 51, 52, 56, and 58	primary bladder adenocarcinoma	NA
Steinestel et al. (2013)^38^	Germany	Case–control	Patients from University of Ulm	74 ± 9.93	89.47%	NA	Biopsy, fixed tissue	PCR	HPV genotypes 6, 11, 16, 18, 31, 33, 35, 39, 45, 51, 52, 53, 56, 58 and 59	UC in situ	normal urothelium/epithelium adjacent to UCIS/CIN III and tumour‐free urothelial tissue samples
Shigehara et al. (2013)^39^	Japan	Case–control	Female patients with primary bladder tumor who underwent transurethral resection (TUR) operation from 1996 to 2010	68.8 (36 – 89)	0	NA	Biopsy, fixed tissue	PCR; ISH; IHC	14 high‐risk HPV types (16, 18, 31, 33, 35, 39, 45, 51, 52, 56, 58, 59, 66, and 68), 5 low‐risk HPV types (6, 11, 42, 43, and 44), and two unknown‐risk types (53 and CP8304)	UC	patients with nonmalignant squamous cell (SC) metaplasia of the urinary bladder
Shaker et al. (2013)^16^	Egypt	Case–control	Patients from the Urology Department at the Theodor Bilharz Research Institute (TBRI) Hospital with bladder lesions	45.5 ± 6.2	88.24%	NA	Biopsy, fixed tissue	ISH	HPV types 6/11 and 16/18	UC; schistosomal‐associated urothelial carcinoma; SQCC	patients subjected to prostatectomy and patients with chronic cystitis
Panagiotakis et al. (2013)^40^	Greece	Case–control	Patients diagnosed with primary urothelial cancer who underwent surgical transurethral resection at the Department of Urology, Asklipeiion General Hospital, V oula, Athens, Greece, between May 2006 and January 2007	75.5 (44–86)	NA	NA	Biopsy, frozen tissue	qPCR	NA	UC	normal bladder mucosa
Chapman‐Fredricks et al. (2013)^41^	USA	Cross‐sectional study	Patients with primary SCC of urinary bladder University of Miami/Jackson Memorial Hospital (Miami, Florida)	NA	57.14%	NA	Biopsy, fixed tissue	ISH; PCR	HPV 16/18, HPV 31, 33, 35, 52, and 58	SCC	NA
Berrada et al. (2013)^42^	Morocco	Case–control	Patients from Urology department of the Military Hospital of Instruction Mohammed Vth in Rabat, Morocco	65 (32–86)	88.37%	NA	Biopsy, frozen tissue	PCR	HPV 16, 31	UC	patients with cystitis
Polesel et al. (2012)^43^	UK	Case–control	Patients above 18 years from an on‐going multi‐centric case ‐control study of TCB in the province of Pordenone between August 2004 and July 2007	NA	83.33%	86.84%	First‐voided urine	PCR	high‐risk and potential high‐risk HPV types (16, 18, 26, 31, 33, 35, 39, 45, 51, 52, 53, 56, 58, 59, 66, 68a, 70, 73, and 82), HPV68 (68b) and two mucosal low‐risk HPV types (HPV6 and HPV11)	TCB	patients admitted for a wide spectrum of acute conditions to the same hospitals where cases had been interviewed
Barghi et al. (2012)^44^	Iran	Cross‐sectional study	Male patients with bladder tumors between February 2004 and February 2007 in Tehran	NA	100%	NA	Biopsy, fixed tissue	PCR	HPV 16/18	TCC	NA
Alexander et al. (2012)^45^	USA and Italy	Cross‐sectional study	Patients from the surgical pathology files of participating institutions, between the years of 1992 and 2011	64 (37–96)	1: 64.28%; 2: 77.78%	NA	Biopsy, fixed tissue	IHC, ISH	HPV genotypes 6 and 11; HPV genotypes 16, 18, 31, 33, 35, 39, 45, 51, 52, 56, 58, and 66	SCC, UC with squamous differentiation	NA
Yavuzer et al. (2011)^46^	Turkey	Cross‐sectional study	Patients with urothelial bladder carcinoma who accepted transurethral resection or cystectomy	61.9 ± 13.4	87.14%	NA	Biopsy, fixed tissue	PCR	18 HPV genotypes, including HPV types 16, 18, 31, 59, 45; 33, 6/11, 58, 52, 56; 35, 42, 43, 44; and 68, 39, 51, 66	UC	NA
Shigehara et al. (2011)^47^	Japan	Case–control	Patients with primary bladder carcinoma underwent transurethral resection between 1997 and 2009 at Kanazawa University Hospital	68.8 (36–89)	80.34%	NA	Biopsy, fixed and frozen tissue	PCR, ISH	21 HPV genotypes, including 14 high‐risk HPV types (16, 18, 31, 33, 35, 39, 45, 51, 52, 56, 58, 59, 66, and 68), 5 low‐risk HPV types (6, 11, 42, 43, and 44), and 2 unknown‐risk types (53 and CP8304)	TCC, SCC, adenocarcinoma and others	patients who had suspected malignant disease but were diagnosed only with inflammatory disorders
Cai et al. (2011)^48^	Italy	Case–control	Patients with non‐muscle invasive bladder cancer (NMIBC) who had undergone transurethral resection of a bladder tumour (TUR‐BT) at the same urological unit between December 2005 and January 2007	73.9 (52–81)	94.87%	0%	early morning, spontaneously voided urine and fresh tissue	PCR	HPV 6, 11, 16, 18, 26, 31, 33, 35, 39, 40, 43, 44, 45, 51, 52, 53, 54, 56, 58, 59, 66, 68, 69, 70, 71, 73, 74, 82	NA	subjects were recruited from among all the patients attending the same urological clinic for other non‐malignant urological diseases over the same period, matched for age, gender and risk factors; all controls are affected by bladder outlet obstruction due to benign prostatic hyperplasia and underwent transurethral resection of the prostate (TUR‐P)
Barghi et al. (2011)^a^^,^ ^49^	Iran	Cross‐sectional study	Patients in Urology and Nephrology Research Center (UNRC), Shahid Beheshti University	61.6 ± 10	87.27%	NA	bladder tissue and urine specimens	PCR	HPV16 and 18 subtypes	TCC	NA
Ben Selma et al. (2010)^50^	Tunisia	Cross‐sectional study	Bladder cancer patients diagnosed in the Department of Pathology, CHU Farhat Hached, Sousse, Tunisia from January 2003 to December 2004; all patients had no previous anogenital HPV‐related lesions, were HIV‐negative, and none of them had undergone organ transplantation	70 (28–99)	87.20%	60%	Biopsy, fixed tissue	PCR	common HPV types 6, 11, 16, 18, 30, 31, 33, 35, 45, 51, and 52; high and intermediate oncogenic risk HPV types 16, 18, 31, 33, 35, 52, and 58; low‐risk oncogenic HPV types 6 and 11	TCC, SCC and adenocarcinoma	NA
Aggarwal et al. (2009)^51^	India	Cross‐sectional study	Patients of urothelial carcinoma of the urinary bladder	56.5 (28–80)	84.85%	NA	Biopsy, fixed tissue	PCR	HPV 16 and 18	UCB	NA
Eslami et al. (2008)^b^^,^ ^52^	Iran	Case–control	NA	Most 51–60	86.49%	NA	Biopsy, fixed tissue	PCR	HPV high risk typing	TCC	non‐neoplastic cases
Moonen et al. (2007)^53^	Netherlands	Cross‐sectional study	Patients in Radboud University Nijmegen Medical Centre who were suspected of having a bladder tumour or were under surveillance for bladder cancer	NA	NA	NA	frozen bladder wash samples	PCR	NA	NA	NA
Helal Tel et al. (2006)^54^	Egypt	Cross‐sectional study	Patients admitted at the Ain‐Shams University hospital	50.8 ± 8.32	89.47%	NA	Biopsy, fixed tissue	ISH	HPV types 6/11, 16/18 and 31/33	TCC, SCC, adenocarcinoma and sarcomatoid carcinomas	NA
Guo et al. (2006)^55^	USA	Cross‐sectional study	Patients with noninvasive squamous lesions (excluding nonkeratinizing metaplasia)	70.09 (48–95)	54.55%	NA	Biopsy, fixed tissue	ISH	HPV 6, 11, 16, 18, 31, 33, 45, and 51	SCC in situ	NA
Youshya et al. (2005)^56^	UK	Cross‐sectional study	Patients with TCC from Royal London Hospital	73 (21– 95)	NA	NA	Biopsy, fixed or frozen tissue	PCR, ISH	HPV types 6, 11, 16, 18, and 40, HPV types 31 and 35, HPV types 33, 34, and 39	TCC	NA
Yang et al. (2005)^57^	USA	Case–control	Patients with schistosomiasis‐associated bladder tumors	NA	NA	NA	tumor biopsy, urine sediment, serum, PBF	mass spectroscopy coupled with competitive PCR	HPV‐16	NA	The tissue controls were DNA samples from normal placentas. The serum and PBF controls were DNA samples we isolated from sera and PBF of anonymous minors not known to be exposed to HPV. The urine sediment controls were DNA samples from normal volunteers.
Escudero et al. (2005)^58^	Spain	Case–control	Patients from Reina Sofia University Hospital (Cordoba, Spain)	61.8 ± 7.3	91.89%	NA	Biopsy, fixed tissue	PCR	HPV 6, 11, 16 and 18	UC	normal urothelium and chronic nonspecific cystitis
Barghi et al. (2005)^59^	Iran	Case–control	Patients with transitional cell carcinoma of bladder who underwent transurethral resection of bladder tissue from October 1999 to May 2002 in Tajrish Shohada medical center	67 ± 10.8	84.75%	54.20%	Biopsy, fixed tissue	PCR	HPV 6, 11, 31 and 33; HPV 16, 18 and 35	TCC	patients with non‐neoplastic disorders
Wang et al. (2004)^60^	USA	Cross‐sectional study	Patients at Washington University Medical Center from the 1989–2002	NA	NA	NA	Biopsy, fixed tissue	PCR	high‐risk HPVs: 16, 18, 31, 33, 35, 39, 45, 51, 52, 53, 56, 58, 59, 66, 68, 70; and low‐risk HPVs: 6, 11, 34, 40, 42, 43, 44, 54, 74	urinary bladder primary small cell carcinomas	NA
Khaled et al. (2003)^61^	Egypt	Cross‐sectional study	Patients with bilharzial bladder cancer who attended the National Cancer Institute, Cairo during the years 1996 through 1997	55 (22– 72)	73.74%	NA	Biopsy, fixed and frozen tissue	PCR	HPV‐6, ‐11, ‐16, ‐18 and ‐33	SCC, TCC, adenocarcinoma, TCC with squamous differentiation, undifferentiated carcinoma	NA
Fioriti et al. (2003)^62^	Italy	Case–control	Patients affected by primary bladder neoplasia who underwent transurethral resection of the tumor (TURB)	64.6 (20–92)	87.50%	NA	Fresh biopsy fragments	PCR	NA	NA	autoptic samples of healthy bladder
Shan et al. (2002)^63^	China	Case‐control	Patients with TCC during 1996‐1998 at the Department of Pathology, Cancer Institute (Hospital), Chinese Academy of Medical Sciences and Peking Union Medical College in Beijing, China	57.4 (27–82)	NA	NA	Biopsy, fixed tissue	PCR	HPV‐18	TCC	normal bladder specimens
Soulitzis et al. (2002)^64^	Greece	Cross‐sectional study	Patients with histologically confirmed bladder cancer from the Department of Urology, University General Hospital of Heraklion, Greece	66.1 ± 11.4	82%	NA	Biopsy, frozen tissue	PCR	HPV types 11, 16, 18 and 33	NA	NA
Westenend et al. (2001)^65^	Netherlands	Cross‐sectional study	Patients with a diagnosis of SCC of the bladder	70 (54– 96)	56.25%	NA	Biopsy, fixed tissue	ISH	HPV 6/11, 16/18 and 31/33/51	SCC	NA
Sur et al. (2001)^66^	South Africa	Cross‐sectional study	Patients with bladder TCCs between the period 1994 and 1996	NA	NA	NA	Biopsy, fixed tissue	NISH, PCR	HPV 6, 11, 16, 18, 31, 33	TCC	NA
Chen et al. (2000)^67^	China	Case–control	Patients with TCC in Linyi Cancer Hospital from 1988 to 1997	58.5 (24–81)	82.67%	NA	Biopsy, fixed tissue	PCR	HPV‐6, ‐11, ‐16, ‐18	TCC	normal bladder mucosa specimen
Yu et al. (1999)^68^	China	Cross‐sectional study	Patients with bladder cancer in the First Hospital of Beijing Medical University from January 1991 to December 1997	62.1 (36–84)	86.54%	NA	Biopsy, fixed tissue	ISH	HPV‐16, ‐18	TCC	NA
Tekin et al. (1999)^69^	Turkey	Case–control	Patients with bladder cancer who patients did not have any evidence of immunosuppression and HPV lesions in the genital or intrauretral areas	NA	NA	NA	Fresh biopsy specimens	PCR	HPV‐16, ‐18	TCC	patients with ureteral stone disease
Simoneau et al. (1999)^70^	Canada	Cross‐sectional study	Patients with Ta/T1 first primary bladder carcinoma from 1990 to 1992	NA	NA	NA	Biopsy, frozen tissue	PCR	HPV type 6, 11, 16, 18 and 33	NA	NA
De Gaetani et al. (1999)^71^	Italy	Cross‐sectional study	Patients with transitional cell papillary carcinoma of the urinary bladder between 1995 and 1997	66.3 ± 19.8	88.37%	NA	Biopsy, fixed tissue	ISH	HPV types 6, 11, 16, 18, 30, 31, 33, 35,45, 51, 52 and types 6, 11, 16, 18, 31, 33, 35, 42, 43, 44, 45, 51, and 56	TCC	NA
Li et al. (1998)^72^	China	Cross‐sectional study	Patients with TCC in Institute of Urological Surgery, Tianjin	59 (29–86)	NA	NA	Biopsy, fixed tissue	PCR	HPV type 6, 11, 16, 18, 31 and 33	TCC	NA
Gazzaniga et al. (1998)^73^	Italy	Cross‐sectional study	Patients with TCC in Cristo Re Hospital of Rome	67 (27–81)	82.86%	NA	Biopsy, frozen tissue	PCR	HPV‐16, ‐18	TCC	NA
Aynaud et al. (1998)^74^	France	Cross‐sectional study	Patients with papillomatous bladder tumors were enrolled during a 6‐month period by 19 urologists belonging to the Collhge Europeen et Francophone d'Urologie Liebrale	68 (30– 85)	79.31%	44.83%	Biopsy, frozen tissue	PCR, southern blot hybridization	HPV‐6,11 and 42, HPV‐16, 18 and 33, HPV‐31, 35 and 39	TCC	NA
Lu et al. (1997)^75^	UK, Egypt	Cross‐sectional study	Patients from Department of Histopathology, Hammersmith Hospital, London, UK, and Urology and Nephrology Center, Mansoura University, Egypt during 1987 ‐ 1994	NA	NA	NA	Biopsy, fixed tissue	ISH	HPV 16 and 18	TCC, SCC and adenocarcinoma	NA
Cooper et al. (1997)^76^	South Africa	Cross‐sectional study	Patients with schistosomiasis associated bladder cancer	47 (29– 72)	72%	NA	Biopsy, fixed tissue	NISH, PCR	HPV 6, 11, 16, 18, 31, and 33	SCC	NA
Chan et al. (1997)^77^	China	Case–control	Patients with papillary transitional cell carcinoma of grades I and II at Queen Mary Hospital in the years 1987–94	NA	NA	NA	Biopsy, fixed tissue	PCR	HPV 6, 11, 16, 18, 31, and 33	TCC	inverted papilloma
Tenti et al. (1996)^78^	Italy	Cross‐sectional study	Patients with TCC who underwent transurethral resection or cystectomy	66.2 ± 23.01	87.34%	NA	Biopsy, fixed tissue	PCR, southern blot hybridization	HPV type 6, 11, 16, 18 and 33	TCC	NA
Ludwig et al. (1996)^79^	Germany	Case–control	Patients were studied prospectively during a 3‐month period for suspicion of bladder cancer, verified by urethrocystoscopy	53 (37–76)	78.13%	NA	Fresh biopsy specimens	PCR	HPV type 6b, 11, 16 and 18	TCC, SCC and adenocarcinoma	chronic cystitis without evidence of malignancy
Lopez‐Beltran et al. (1996)^80^	Spain	Cross‐sectional study	Patients with TCC of the urinary bladder received at Reina Sofia University Hospital (Cordoba, Spain)	66.57 ± 1.17	81.58%	NA	Biopsy, fixed tissue	PCR	HPV types 6, 11, 16 and 18	TCC	NA
Boucher et al. (1996)^81^	UK	Cross‐sectional study	NA	68 (44–88)	78.18%	NA	Biopsy, fixed tissue	DNA hybridization and dot‐blot analysis	HPV types 6, 11 and 16	TCC, SCC	NA
Kim et al. (1995)^82^	Korea	Cross‐sectional study	Patients in Yonsei Medical Center	NA	NA	NA	Biopsy, fixed tissue	PCR and dot‐blot hybridization	HPV types 6, 11, 16, 18, 31 and 33	TCC	NA
Kamel et al. (1995)^83^	Finland	Cross‐sectional study	Patients with bladder carcinomas diagnosed during the years 1987‐1992 in Oulu University Central Hospital	77.6 ± 8.5 for females; 68.5 ± 10.1 for males	63.83%	NA	Biopsy, fixed tissue	ISH	HPV types 6, 11, 16, 18, 31 and 33	TCC, SCC	NA
Gopalkrishna et al. (1995)^84^	India	Cross‐sectional study	Male patients in Urology Clinics, K.G. Medical College Hospital, Lucknow	NA	100%	NA	Biopsy, fixed tissue	PCR, ISH	HPV 16	TCC	NA
Smetana et al. (1995)^85^	Israel	Case–control	Patients with transitional cell carcinoma in Jewish population in Israel	NA	NA	NA	Biopsy, fixed tissue	PCR; peroxidase anti‐peroxidase (PAP) method for HPV capsid antigen; ISH	HPV 6/11,16/18,31/33/35	TCC	nontumoral material of the bladder or post mortem specimens
Chang et al. (1994)^86^	Finland	Cross‐sectional study	Patients with invasive TCC from 1966 to 1987	32–81	83.33%	NA	Biopsy, fixed tissue	PCR, ISH	HPV ‐6, ‐11, ‐16, ‐18, ‐31, ‐33, ‐35, ‐39, ‐40, ‐45, ‐51, ‐59	TCC	NA
Aglianò et al. (1994)^87^	Italy	Case–control	NA	NA	82.61%	NA	Biopsy, fixed tissue	PCR	HPV 16 and 18	TCC	non‐neoplastic normal urinary sample
Mincione et al. (1994)^88^	Italy	Cross‐sectional study	NA	NA	NA	NA	Biopsy, fixed tissue	ISH	HPV 6/11,16,18,31/33/51	TCC	NA
Saltzstein et al. (1993)^89^	USA	Cross‐sectional study	NA	NA	NA	NA	Biopsy, fixed and fresh tissue	PCR, southern blot	HPV 6, 11, 16, 18, 31, 33	TCC	NA
Furihata et al. (1993)^90^	Japan	Cross‐sectional study	Patients with transitional cell carcinoma of the urinary bladder between 1981 and 1992 at the Department of Urology of Kochi Medical School and the Division of Urology of Kochi Takasu Hospital	49–92	76.67%	NA	Biopsy, fixed tissue	ISH	HPV types 16, 18, and 33	TCC	NA
Yu et al. (1993)^91^	China	Case–control	NA	NA	NA	NA	Fresh specimens	PCR	HPV‐16, ‐18	TCC	patients with hyperplasia of prostate
Wilczynski et al. (1993)^92^	USA	cross‐sectional study	NA	NA	NA	NA	Biopsy, frozen tissue	PCR, ISH, southern blot	HPV types 6, 16, 18, and 31	SCC	NA
Shibutani et al. (1992)^93^	USA	Cross‐sectional study	Consecutive patients with transurethrally resected bladder tumors at Pennsylvania Hospital	64 (34– 89)	60.87%	NA	Fresh specimen	southern blot	HPV types 6/11, 16/18, and 31/33	TCC	NA
Knowles et al. (1992)^94^	UK	Cross‐sectional study	NA	NA	NA	NA	Biopsy, frozen tissue	PCR, southern blot	HPV 1, 6, 8, 11, 13, 16, 18, 30, 31, 32, 33, 45 and 51	TCC, SCC and adenocarcinoma	NA
Chetsanga et al. (1992)^95^	Zimbabwe	Cross‐sectional study	Patients with transitional cell carcinoma of the urinary bladder	NA	NA	NA	Biopsy, frozen tissue	PCR and dot‐blot hybridization	HPV 6b, 11, 16, 18 or 33	TCC	NA
Anwar et al. (1992)^96^	Japan	Case–control	Patients from Fukui Medical School, Japan	68	70.83%	NA	Biopsy, fixed tissue	PCR, ISH, southern blot and dot‐blot hybridization	HPV 6, 16, 18, and 33	TCC, SCC	autopsy material from patients who had died of causes other than urogenital malignant neoplasms
Bryant et al. (1991)^97^	UK	Case–control	Patients in Princess of Wales Hospital	NA	NA	NA	Biopsy, fixed tissue	ISH	HPV types 6b, 11, 16 and 18	TCC	Dysplasia and benign bladder tissues
Kulski et al. (1990)^98^	Australia	Cross‐sectional study	Patients in Sir Charles Gairdner Hospital, Queen Elizabeth Medical Centre, and King Edward Memorial Hospital for Women	NA	NA	NA	Biopsy, fixed tissue	ISH	HPV‐6,11,16,18	NA	NA

Abbreviations: HPV, human papilloma virus; IHC, immunohistochemistry; ISH, in situ hybridization; NA, not available; NISH, nonisotopic in situ hybridization; PBF, peripheral blood fraction; PCR, polymerase chain reaction; qPCR, quantitative real‐time PCR; RISH, mRNA in situ hybridization; SCC, squamous cell carcinoma; SQCC, schistosomal squamous cell carcinoma; TCC, transitional cell carcinoma; TUBC, transitional urothelial bladder carcinoma; UC, urothelial carcinoma; UCB, urothelial carcinoma of bladder;.

^a^ Meeting abstract from European Association of Urology (EAU) 7th South Eastern European Meeting.

^b^ Poster Presentations from 13th International Congress on Infectious Diseases Abstracts.

### 2.4 Literature quality assessment

The evaluation criteria for an observational study of the Agency for Healthcare Research and Quality (AHRQ) were adapted to evaluate the quality of cross‐sectional studies.^99^ On the other hand, the Newcastle–Ottawa scale (NOS) was used to assess the quality of case–control studies.^100^ Agency for Healthcare Research and Quality evaluation criteria for an observational study consists of 11 items. Every item of AHRQ criteria was answered as yes, no, or not reported. The items with “yes” scored one, and the items with “no” and “not reported” scored zero. The scores between 8 and 11 were regarded as high quality, 4 and 6 as moderate quality, and 0 and 3 as low quality. The NOS assigns a maximum of nine points to eight items in three categories: selection, comparability, and outcome. A study can be awarded a maximum of one point for each numbered item within the selection and outcome categories. On the other hand, a maximum of two points can be given to items in the comparability category. A study was regarded to be of high quality if it had a score of more than six. Two researchers, Z. X. Y. and Z. N., conducted the risk of bias and literature quality assessment, and the discrepancies were resolved by the consensus of the third author, A. Y.

### 2.5 Data synthesis and statistical analysis

This study estimated the prevalence of overall and type‐specific HPV and 95% CI using the size and number of HPV‐positive infections in BCa patients using Random Effects (RE) models or Fixed Effects (FE) models.^101^ Furthermore, the pooled odds ratio (pOR) was calculated with 95% CI using the data obtained from case–control studies to assess the risk of HPV infection on BCa. The pooled risk ratio (pRR) with 95% CI was also calculated to assess the association between HPV infection and the prognosis of BCa. The heterogeneity between studies was also analyzed using the standard Cochrane Chi‐square *χ* ^2^ (Cochrane's *Q*) test with a significance level of *α* = 0.10 and the *I* ^*2*^ test.^102^ The *I* ^*2*^ describes the percentage of variation across studies, and an *I* ^*2*^ statistic value of ≥50% indicates considerable heterogeneity.^103^ The L'Abbé and Galbraith plots were used to display the heterogeneity of included studies visually. Subgroup analysis stratified by parameters such as continent, histological type of BC, and detection method was performed to find out the potential source of heterogeneity. The study also performed meta‐regression using parameters such as age, percentage of male patients, and smoking rate, which could be latent confounders among the studies. The publication bias in observational studies was determined using both the Begg's^104^ and Egger's^105^ tests. A contour‐enhanced funnel plot was utilized to determine other causes of publication bias by examining the symmetry of the plot. Lastly, the study did a sensitivity analysis and cumulative meta‐analysis by adding or omitting included studies stepwise and also applied the trim and fill method to evaluate the effect of publication bias.^106^ A filled forest plot was constructed to preclude the publication bias on pOR and pRR. This study used the R software version 4.2.0 with the “meta” package^107^ and “metagen” command^108^ for data processing and statistical analysis. All the p values were two‐sided, and a *p* < 0.05 was considered significantly different.

### 2.6 MR

The data resources were obtained from MRC IEU OpenGWAS (https://gwas.mrcieu.ac.uk/; version: v6.5.2‐2022‐04‐11), developed at the MRC Integrative Epidemiology Unit at the University of Bristol.^109^ The GWAS ID of summary‐level data included in this study were: prot‐c‐2623_54_4 (E7 protein of HPV16), prot‐c‐2624_31_2 (E7 protein of HPV18), finn‐b‐C3_BLADDER_EXALLC (malignant bladder tumor, cases = 1115, noncases = 174 006), finn‐b‐CD2_BENIGN_BLADDER_EXALLC (benign bladder tumor, cases = 109, noncases = 180 709), ieu‐b‐4874 (malignant bladder tumor, cases = 1279, noncases = 372 016), ukb‐d‐C67 (malignant bladder tumor, cases = 1554, noncases = 359 640), as shown in Supporting Information: Table S8.

Supporting Information: Table S9 shows the 36 single‐nucleotide polymorphisms (SNPs) from four genome‐wide association studies (GWAS). These SNPs were considered as instrumental variables to evaluate the causality of HPV E7 protein (*p* < 5 × 10^−5^, *r* ^2^ < 0.001 and clump distance >10 000 kb). Five MR methods were used: Weighed median regression, Inverse variance weighting (IVW), Mendelian randomization‐Egger (MR Egger), Simple mode, and Weighed mode. The IVW method with RE was the main statistical model. This study applied the SNP‐specific Wald ratio to evaluate the causality. The pleiotropy test tested the pleiotropic effects, and *p* ˃ 0.05 was regarded as having no pleiotropic effects. Heterogeneity was tested using the Cochrane Q value. Supporting Information: Tables S10 and S11 show the detected results during the pleiotropy and heterogeneity tests. Detailed information on SNPs for HPV E7 protein and bladder tumor is shown in Supporting Information: Table S12. This study used the fixed‐effect meta‐analysis method to combine the estimated values from different data sources.

All *p* values were two‐tailed, and the R software (version 4.2.0) with the Two Sample MR package^110^ was used to perform all the statistical analyses.

## 3 RESULTS

This study retrieved 546 publications from electronic databases and other sources. However, after applying the inclusion and exclusion criteria in Figure 1, 466 articles were excluded, and 80 were included in this systematic review and meta‐analysis study. Of the 466 excluded articles, 99 duplicates were removed using automatic tools in the Endnote application, and 52 were removed using artificial identification by reviewers. On the other hand, 248 articles were removed after reading the title and abstract, and 20 articles were excluded for not having full text or original data. After reading the full text, 37 case reports were excluded, and four publications were excluded because they included patients who received organ transplants or immunosuppressed patients. Four articles were removed because they had duplicated patients with other articles and two articles were excluded for not having a primary bladder tumor.

### 3.1 Characteristics of included studies and patients

The characteristics of the 80 articles included in this study are shown in Table 1. Of the 80 articles, two were meeting abstracts,^49^, ^52^ 27 were case–control studies, and 53 were cross‐sectional studies. The studies were conducted in 26 countries and five continents including Europe, North America, Asia, Africa, and Oceania. Two studies^45^, ^75^ included patients from more than one country. There were 6065 BC patients, and the number of BCa patients varied from five to 689 in each study. The match group in case–control studies was varied, including patients with benign bladder pathologies, tumor‐free urothelial tissue samples, patients subjected to prostatectomy, and normal bladder mucosa specimens. Seven studies contained follow‐up information on patients with BCa, and the follow‐up duration ranged from 18 months to 10 years. The outcome of interest consisted of recurrence, progression, and death of patients with BCa.

In these studies, most patients were more than 60 years, and the percentage of male patients was greater than 80%. It was found that only one study focused on female patients with BCa.^39^ It was also found that the smoking rate of the patients in most studies was greater than 60%. Unlike most studies whose samples for HPV detection were fixed or frozen tissue biopsy, few studies used first‐morning urine and urethral swab samples for HPV detection. The detection method used in most studies included PCR and ISH. The review of the literature found that TCC and SCC were the most common histological types of BC. However, TCC was discussed in most studies. On the other hand, it was found that SCC was associated with schistosomes, especially in studies in Egypt. In total, 57 studies detected the specific HPV subtypes in BC patients, and the detailed information is shown in Supporting Information: Table S2.

### 3.2 Quality assessment of the included studies

This study adopted the Agency for Healthcare Research and Quality (AHRQ) evaluation criteria to evaluate the quality of cross‐sectional studies, as shown in Supporting Information: Table S3. The Newcastle–Ottawa scale (NOS) was also adopted to assess the quality of case‐control studies, as shown in Supporting Information: Table S4. The use of AHQR and NOS showed that all the articles included in this study were of high or moderate quality.

### 3.3 The prevalence of HPV in bladder cancer cases

**Figure 2:**
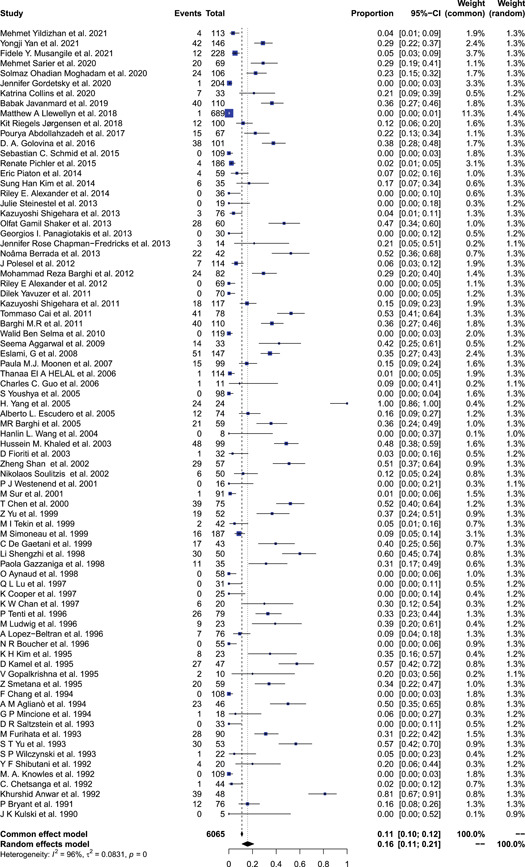
Forest plot for the prevalence of HPV in bladder cancer cases. Pooled prevalence and 95% confidence intervals of HPV infection, using a random‐effect model. HPV, human papillomavirus.

**Figure 3:**
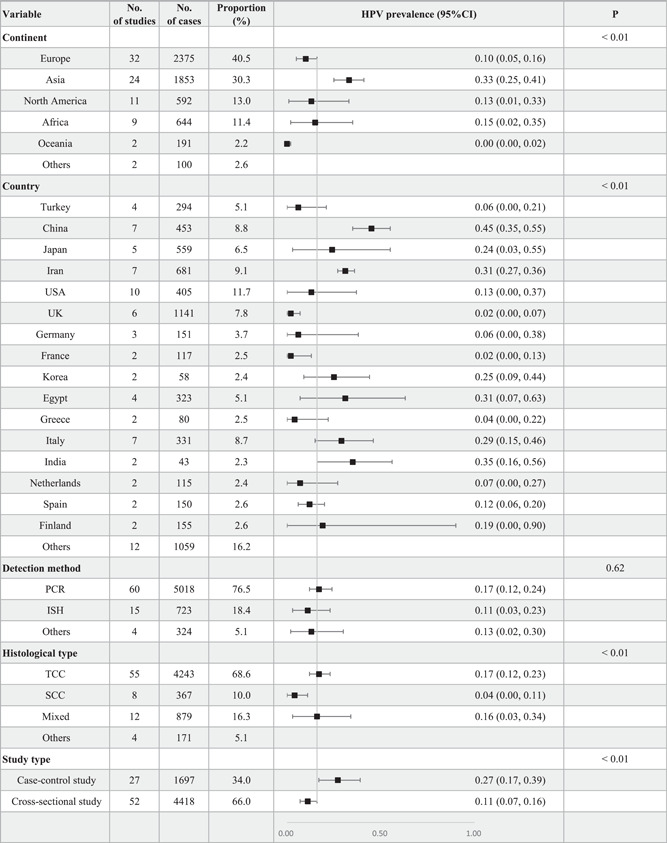
Subgroup analyses for HPV prevalence in bladder cancer cases stratified by continent, country, detection method, histological type, and study type. CI, confidence interval; ISH, in situ hybridization; No., number; PCR, polymerase chain reaction; SCC, squamous cell carcinoma; TCC, transitional cell carcinoma.

The 80 studies had 6065 patients with BCa, in which the prevalence of HPV ranged from 0% to 100%, and the overall HPV prevalence was 16% (95% CI: 11%–21%, *I* ^2^ = 96%, RE model), as shown in Figure 2. The results obtained in this study were consistent with the previous study.^19^ Subgroup analyses stratified by continent found that most HPV cases were in Europe (40.5%) and Asia (30.3%), as shown in Figure 3. The results in Figure 3 also showed the prevalence of HPV was highest in Asia (33%, 95% CI: 25%–41%) and Africa (15%, 95% CI: 2%–35%). The prevalence of HPV in Europe (10%, 95% CI: 5%–16%) was relatively low compared to other continents. The study found that the prevalence of HPV was highest in China (45%, 95% CI: 35%–55%) and India (35%, 95% CI: 16%–56%). In addition, this study found that most studies used qualitative PCR to detect HPV. Other methods such as quantitative real‐time PCR, ISH, and southern bot were also applied. It was found that studies that used PCR showed the highest prevalence of HPV (17%, 95% CI: 12%–24%) and seemed the most sensitive.

The study also investigated the prevalence of HPV in different histological subtypes of BCa, as shown in Figure 3. In most studies, TCC was the primary histological subtype and showed a detection rate of 17% (95% CI: 12%–23%). The SCC subtype was the second common histological subtype and exhibited a detection rate of 4% (95% CI: 0%–11%). The prevalence of HPV in studies comprising mixed histological subtypes was similar to TCC (16%, 95% CI: 3%–34%) because TCC was common in these studies. On the other hand, the prevalence of HPV in case–control and cross‐sectional studies was 27% (95% CI: 17%–39%) and 11% (95% CI: 7%–16%), respectively.

**Figure 4:**
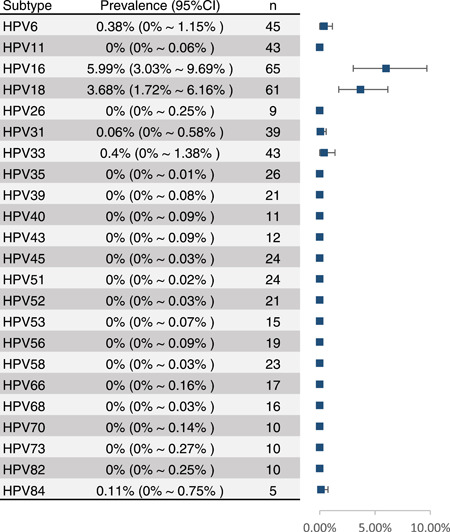
Subgroup analyses for HPV prevalence in bladder cancer cases stratified by HPV subtypes. CI, confidence interval; HPV, human papilloma virus.

The results in Figure 4 show the 23 HPV subtypes (HPV 6, 11, 16, 18, 26, 31, 33, 35, 39, 40, 43, 45, 51, 52, 53, 56, 58, 66, 68, 70, 73, 82, 84) detected in the included studies. The study found that the prevalence of HPV‐16 was highest at 5.99% (95% CI: 3.03%–9.69%), and HPV‐18 was second at 3.68% (95% CI: 1.72%–6.16%). The prevalence of high‐risk HPV (HPV‐16, 18, 31, and 33) was significantly higher than that of low‐risk HPV (HPV‐6, 11 et.). The study performed subgroup analyses for the prevalence of high‐risk HPV subtypes, and eight studies shown in Supporting Information: Figure S1 were included. The study found that the overall prevalence of high‐risk HPV was 29% (95% CI: 6%–60%). In addition, the study found that the prevalence of high‐risk HPV was significantly different in the continent, histological subtypes, and study types.

A contour‐enhanced funnel plot was constructed because the results found that the articles included in this study had significant heterogeneity. However, the result in Supporting Information: Figure S1A shows that a good symmetry of the plot was not found. On the other hand, the Galbraith plot exhibited a relatively high publication bias, with many studies not located between the dashed lines, as shown in Supporting Information: Figure S2B. Therefore, the trim‐and‐fill method was conducted to fill up the missing studies due to publication bias. The funnel plot showed good symmetry after filling, as shown in Supporting Information: Figure S2C. The filled forest plot showed an HPV infection rate of 5% (95% CI: 2%‐9%), which was lower than the original result shown in Supporting Information: Figure S3A. The cumulative meta‐analysis in Supporting Information: Figure S3B and sensitivity analysis in Supporting Information: Figure S3C showed a relatively stable result. This study used Egger's test to identify publication bias. Egger's test found significant publication bias (*t* = 3.52 and *p* = 0.0007). However, Begg's test did not show a significant publication bias (*z* = 1.14, *p* = 0.2529).

Besides, meta‐regression was also performed to find out the latent confounding variables. The study used the percentage of male patients and the smoking rate to construct the univariate meta‐regression model, as shown in Supporting Information: Figure S4. However, the study found no significant association between the prevalence of HPV and the percentage of male patients (*p* = 0.2153) and the smoking rate (*p* = 0.8540), as shown in Supporting Information: Table S5.

### 3.4 The association between HPV infection and bladder cancer risk

**Figure 5:**
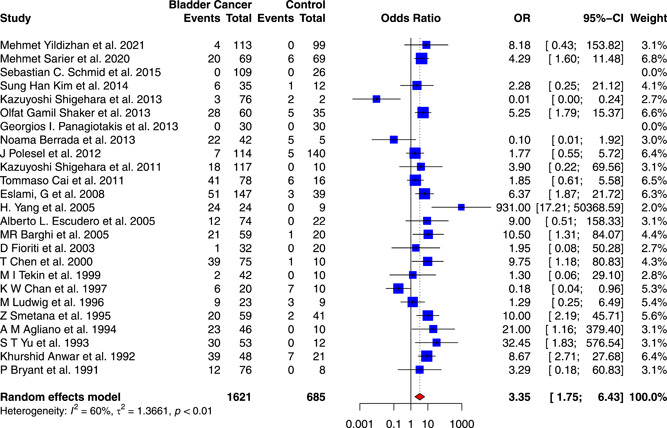
Forest plot for the association between HPV infection and bladder cancer risk. Pooled odds ratios and 95% confidence intervals of bladder cancer risk associated with HPV infection, using the random‐effects model. HPV, human papilloma virus.

**Figure 6:**
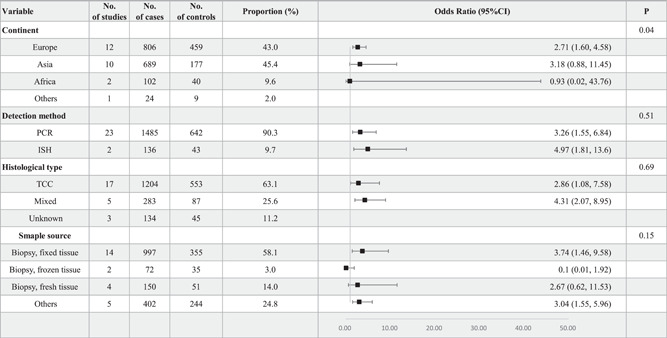
Subgroup analyses for OR of bladder cancer stratified by continent, detection method, histological type and sample source.

A total of 25 case–control studies comprising 1621 BCa patients and 685 used were included to investigate the association between HPV infection and the risk of BCa. Two of these studies^33^, ^40^ were automatically excluded because they lacked HPV‐positive patients. The forest plot in Figure 5 showed that HPV infection was significantly connected with the increased risk of BCa, with a pOR of 3.35 (95% CI: 1.75–6.43, *I* ^2^ = 60%, RE model). A subgroup analysis stratified by continent, detection method, histological subtype, and sample source was also performed, as shown in Figure 6. The results found an increased risk of BCa for HPV‐positive patients in Asia and Europe, but only patients in Europe exhibited a significant difference (OR = 2.71, 95% CI: 1.60–4.58). There did not show significant heterogeneity between different detection methods. Both PCR (OR = 3.26, 95% CI: 1.55–6.84) and ISH (OR = 4.97, 95% CI: 1.81–13.6) exhibited a significantly increased risk of BCa. The TCC subtypes showed a 2.86‐fold risk of BCa (95% CI: 1.08–7.58), and the pOR was 4.31 (95% CI: 2.07–8.95) in patients with mixed histological subtypes (TCC, SCC, and others). Similarly, a significantly increased risk of BCa was found in the biopsy, fixed tissue, and other sample sources (first‐morning urine and urethral swab sample).

A funnel plot shown in Supporting Information: Figure S5A was drawn to identify the heterogeneity of the included studies. The funnel plot showed good symmetry, with only a few publications outside the dashed lines. No missing studies were filled after the fill and trim method, as shown in Supporting Information: Figures S5B and S5C. The Galbraith plot in Supporting Information: Figure S5D exhibited a low publication bias, and the L'Abbé plot in Supporting Information: Figure S5E showed that most included studies supported the increased risk of HPV infection on BCa. Moreover, the study did not identify a significant publication bias based on Begg's test (*z* = −0.66, *p* = 0.5091) and Egger's test (*t* = −0.12, *p* = 0.9092). In addition, the cumulative meta‐analysis and sensitivity analysis were performed by stepwise addition and deletion of studies. There were no observable changes in the POR, as shown in Supporting Information: Figures S6A and S6B.

In univariate meta‐regression, the study found that the percentage of male patients was significantly associated with the increased risk of BCa (*p* = 0.0078) and might serve as a latent confounding factor. On the other hand, the models constructed based on age (*p* = 0.5547), smoking rate (*p* = 0.5918) and the publication year (*p* = 0.3295) had no statistical significance, as shown in Supporting Information: Table S6 and Figure S7.

### 3.5 The interaction between HPV infection and bladder cancer prognosis

**Figure 7:**
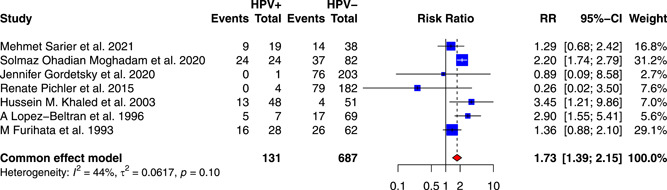
Forest plot for the association between HPV infection and bladder cancer prognosis. Pooled relative risks and 95% confidence interval for the prognosis of bladder cancer associated with HPV infection, using the common‐effects model. HPV, human papilloma virus.

**Figure 8:**
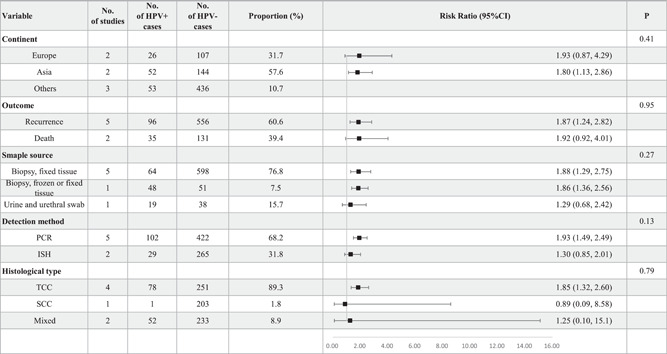
Subgroup analyses for RR of bladder cancer progression stratified by continent, outcomes, detection method, histological type, and sample source.

In this study, seven studies with follow‐up information were collected to investigate the interaction between HPV infection and the prognosis of BCa. Five of the seven studies used recurrence as the outcome event, while two studies used death. The study combined recurrence and death as the outcome for analysis and defined it as progression. In total, 131 HPV‐positive BCa patients and 687 HPV‐negative BCa patients were included in this study. The results showed that HPV infection was significantly connected with an increased risk of progression of BCa, with a pRR of 1.73 (95% CI: 1.39–2.15, *I* ^2^ = 44%, FE model) (Figure 7). In subgroup analyses shown in Figure 8, the study found a significantly increased risk of BCa progression in HPV‐positive patients in Asia (RR: 1.80, 95% CI: 1.13–2.86). The results were similar for patients when using recurrence as the outcome (RR: 1.87, 95% CI: 1.24–2.82). A significantly increased risk of BCa progression with HPV infection was also shown in fixed tissue (RR: 1.88, 95% CI: 1.29–2.75) and frozen or fixed tissue (RR: 1.86, 95% CI: 1.36–2.56). Besides, a significantly increased risk of BCa progression with HPV infection was revealed when HPV DNA was detected using PCR (RR: 1.93, 95% CI: 1.49–2.49) and in patients with TCC subtype (RR: 1.85, 95% CI: 1.32–2.60). And this study did not find a statistically significant difference in all these subgroups.

The funnel plot did not show good symmetry, as shown in Supporting Information: Figure S8A. Therefore, the trim and fill method was used to eliminate the latent publication shown in Supporting Information: Figures S8B and S8C. Two missing publications were filled and yielded a pRR of 1.95 (95% CI: 1.43–2.66), which indicated that the publication bias had weakened the effect of HPV infection on the progression of BCa. The cumulative meta‐analysis shown in Supporting Information: Figure S8D and sensitivity analysis shown in Supporting Information: Figure S8E were also performed, and no observable changes occurred in the pRR. The Galbraith plot exhibited a low publication bias as shown in Supporting Information: Figure S8F. On the other hand, the L'Abbé plot showed that most included studies agreed on the positive effect of HPV infection on the progression of BCa, as shown in Supporting Information: Figure S8G. Besides, the study did not identify a statistically significant publication bias based on Begg's test (*z* = −1.05, *p* = 0.2931) and Egger's test (*t* = −0.95, *p* = 0.3875).

In univariate meta‐regression, the study found the prevalence of HPV‐18 was negatively associated with the risk of BCa (*p* = 0.0326), while the models constructed based on follow up duration (*p* = 0.3950), publication year (*p* = 0.6786), age (*p* = 0.4156), the percentage of male patients (*p* = 0.8388), smoking rate (*p* = 0.0867) and HPV‐16 prevalence (*p* = 0.5226) all lacked statistical significance, as shown in Figure S9 and Table S7.

### 3.6 The meta‐analysis based on MR method

**Figure 9:**
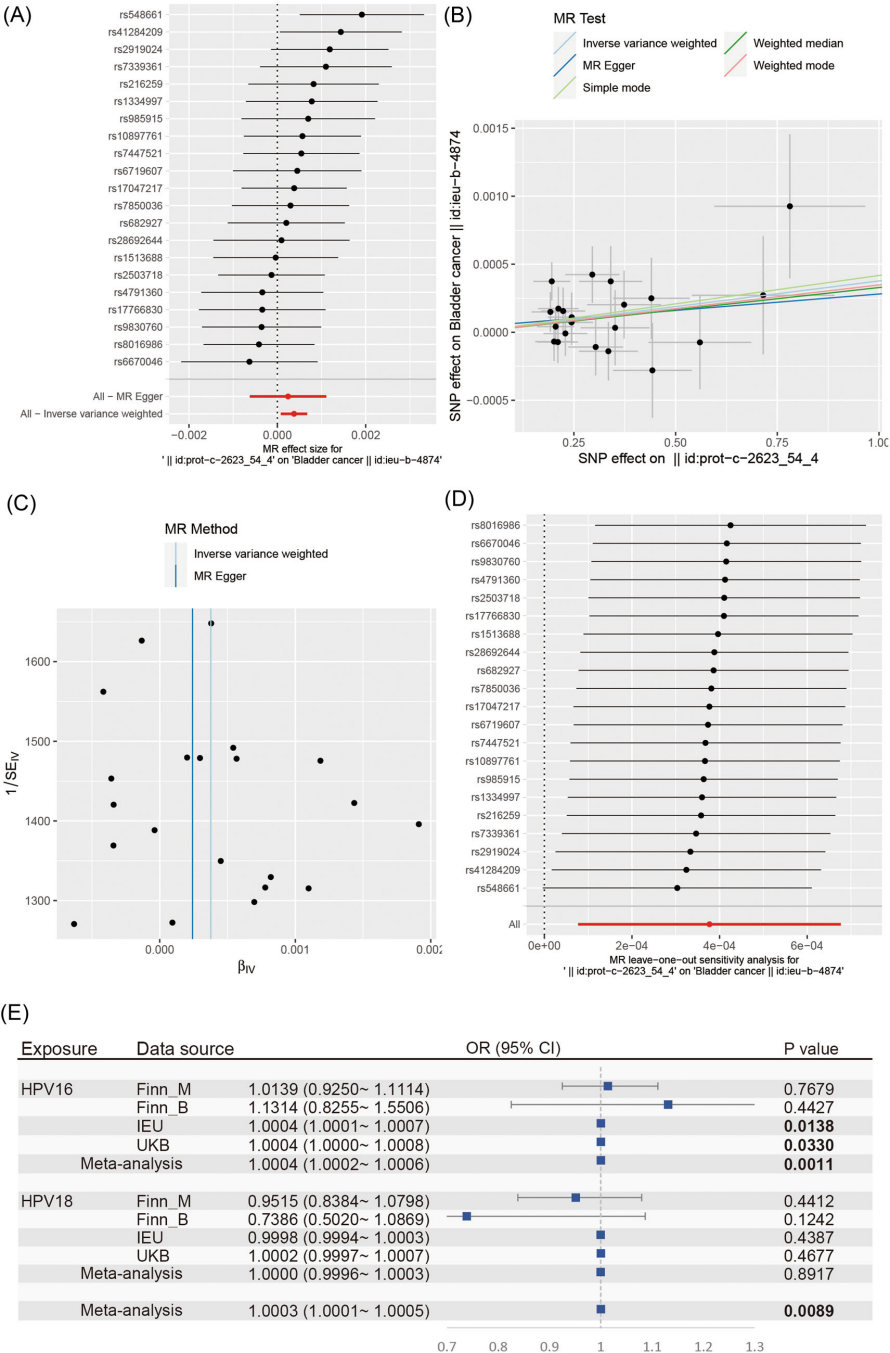
The 2‐sample Mendelian randomization (MR) analysis. (A) Forest plot for the 2‐sample MR analysis representing the causal estimate of HPV‐16 E7 protein for bladder cancer. Circles alongside each SNP represent the causal estimate of each instrumental variable separately, and the lowest two circles show multiple‐instrument MR analysis using Egger regression and inverse‐variance weighted methods. Horizontal lines denote 95% CIs. IVW, inverse variance weighted; SD, standard deviation. (B) Scatter plot for causal effects of HPV‐16 E7 protein on bladder cancer. Slope of the straight line indicates the magnitude of causal association. IVW indicates inverse‐variance weighted; MR, Mendelian randomization. (C) Funnel plots for overall heterogeneity of MR estimates for the effect of HPV 16 E7 protein on bladder cancer. IVW, inverse‐variance weighted. (D) Leave‐one‐out sensitivity analysis of bladder cancer to investigate the possibility of causal association driven by a particular SNP. Each black point represents an inverse variance weighted method for estimating the causal effect of HPV 16 E7 protein on bladder cancer, excluding that particular instrumental variable from the analysis. Redpoint represents the estimate using all instrumental variables. Horizontal lines denote 95% confidence intervals. OR, odds ratio; SD, standard deviation. (E) Meta‐analysis for results of MR studies using different bladder cancer datasets including finn‐b‐C3_BLADDER_EXALLC, finn‐b‐CD2_BENIGN_BLADDER_EXALLC, ieu‐b‐4874, ukb‐d‐C67.

The data source for this two‐sample MR study was shown in Supporting Information: Table S8. Supporting Information: Tables S10 and S11 show the detected results during the pleiotropy and heterogeneity tests. The association for each genetic instrument variable with BCa is presented in Supporting Information: Table S9. Supporting Information: Table S12 shows the causal association between the genetically predicted HPV E7 protein level and the risk of developing BCa in patients from the ieu‐b‐4874 dataset. The study found that the exposure of HPV‐16 E7 protein was significantly positively associated with the increased risk of BCa using the IVW method (OR per unit increase in protein level = 1.000377038, 95% CI: 1.000076826–1.00067734), as shown in Figure 9A,B. Other MR methods for HPV‐16 E7 protein exposure also exhibited directionally similar estimates although lack statistical significance, as shown in Supporting Information: Table S12 and Figure 9B. The symmetry of the funnel plot in Figure 9C shows the directional horizontal pleiotropy of the SNPs. However, it was hard to assess the symmetry of the funnel plot due to the limited number of genetic instruments. The MR‐Egger intercepts showed no evidence for significant directional pleiotropy (*p* = 0.7473), as shown in Supporting Information: Table S10. This result indicated no directional pleiotropic effects existed between HPV 16 E7 protein exposure and BCa.

The study also performed the leave‐one‐out analysis to exclude the influence of individual SNPs on the overall causal estimate by the stepwise removal of each SNP and repeating the MR analyses. The leave‐one‐out analysis showed a relatively stable outcome after removing each SNP, as shown in Figure 9D. A meta‐analysis of the results of MR studies using different BCa datasets was also conducted, as shown in Figure 9E. The results showed that the exposure to HPV‐16 and 18 E7 protein was positively associated with increased risk of BCa (HPV‐16 E7 protein: IVW OR per unit increase in protein level = 1.0004 [95% CI: 1.0002–1.0006], *p* = 0.0011; HPV 18 E7 protein: IVW OR per unit increase in protein level = 1.0003 [95% CI: 1.0001–1.0005], *p* = 0.0089).

## 4 DISCUSSION

An early meta‐analysis published in 2011^19^ investigated the relationship between HPV infection and BCa risk, however, studies have not conclusively elucidated othis association. The previous meta‐analysis only investigated HPV prevalence in BCa cases and the interaction between HPV infection and BCa risk, but did not investigate the role of HPV infection in BCa prognosis. Therefore, we assessed the relationship between HPV infection and BCa risk via both meta‐analysis and two‐sample MR analysis. Eighty studies were included in this study, including 27 case–control and 53 cross‐sectional studies.

A total of 6065 BCa cases were included in this study, in which the prevalence of HPV ranged from 0% to 100%, yielding a pooled HPV prevalence of 16% (95% CI: 11%–21%), consistent with the previous meta‐analysis.^19^ HPV infection was significantly associated with increased risk of BCa (OR = 3.35), and poorer prognosis of BCa patients (RR = 1.73). Findings from two‐sample MR analysis showed significant interactions between HPV E7 protein exposure and BCa (HPV‐16 E7 protein: IVW OR per unit increase in protein level = 1.0004, *p* = 0.0011; HPV‐18 E7 protein: IVW OR per unit increase in protein level = 1.0003, *p* = 0.0089), implying that this interaction represents a causal effect of HPV infection.

BCa is the 10th most common cancer worldwide with high morbidity and mortality rates. Smoking and exposure to several chemicals are known risk factors for BCa and it is important to identify the latent risk factors to prevent BCa. HPV is a family of sexually transmitted DNA‐based viruses that infect the stratified squamous epithelia, and more than 150 HPV subtypes have been identified,^15^, ^111^ of which more than 35 subtypes have specific tropism for the genitourinary tract in both genders.^16^ We found an overall HPV prevalence of 16% in BCa cases, and the main subtype was HPV‐16 (5.99%), followed by HPV‐18 (3.68%). Since most patients with BCa were male, this indicated HPV infection in BCa, especially in male patients, should not be neglected. The prevalence of HPV was highest in Asia (33%) and Africa (15%), exhibiting a moderate geographical variation that is due to differences in genetic factors, sexual behaviors, ethnic and cultural differences, and other unknown risk factors. Moreover, HPV prevalence was significantly associated with detection methods, among which PCR showed the highest prevalence (17%). Technically, PCR has a high sensitivity for HPV detection and most studies use this approach for viral detection. Almost all HPV detection methods rely on the assessment of viral nucleic acids, especially DNA. Southern blot and Northern blot hybridizations were early methods for HPV DNA and RNA detection, respectively. These methods require large amounts of detection material and are inefficient, and time‐consuming. Besides, they are not suitable for the detection of formalin‐fixed tissue samples. In contrast, PCR can amplify target DNA for detection and make it easier to read out. Reverse‐transcriptase (RT)‐PCR can amplify messenger RNA and generate complementary DNA for detection. Moreover, in situ hybridization involves an amplification step, but it amplifies the signal instead of the target.^112^ Therefore, heterogeneities of HPV testing methods across countries and studies are largely attributed to different sample types and available scientific research facilities. Furthermore, there was significant diversity in histological types of BCa. The highest prevalence of TCC is because TCC was the major histological subtype in most studies.

Smoking is an acknowledged risk factor for bladder cancer and has been highly associated with higher incidences of HPV infection (OR = 1.5, 95% CI: 1.4‐1.7), which was dose‐dependent and more significant for female patients (OR = 2.0, 95% CI: 1.8–2.3) and high‐risk HPV subtypes.^113^ These effects might be due to nicotine, the immunosuppressive component in cigarettes,^114^ and metaplasia as well as DNA damage due to smoking.^115^, ^116^ In the meta‐regression analyses, we did not find a statistically significant association between HPV prevalence and smoking rate (*p* = 0.8540). Differences in these outcomes are because: First, smoking status was not accurate in some studies because of information bias, and the duration as well as smoking dose were unknown in most studies, which was significantly associated with HPV prevalence. Second, most of the included patients were male, however, HPV prevalence was higher among female smokers. Finally, only a limited number of studies reported on the smoking status of patients, therefore, the result should be interpreted with caution.

Globally, HPV infections account for 630 000 new cancer cases per year, among which 83% are cervical cancer cases.^117^ Mechanistically, when HPV DNA is inserted into the host genome,^118^ HPV‐related E6 and E7 proteins inactivate or degrade suppressor gene‐associated proteins (p53 and RB1) to drive BCa tumorigenesis.^59^, ^119^ High‐risk HPV E6 proteins can activate telomerase reverse transcriptase (TERT) and telomerase, which are important for the maintenance of telomere lengths and are vital for cell immortality.^120^ Besides, the E6 protein of HP‐16, HPV‐8, and HPV‐1 can bind and inhibit the DNA repair protein (XRCC1) to prevent DNA repair after breakage and allow the accumulation of mutations in the host genome.^121^ Moreover, E6 can also mediate the activation of EGF and MAPK signaling pathways to promote cell growth.^122^ The E7 protein can result in mistakes in centrosome duplication by influencing the activities of cyclin E/cyclin‐dependent kinase 2 (CDK2) complexes, leading to genomic instability.^123^ We established that HPV infection increased the risk of BCa by 3.35‐fold, and this effect was not influenced by detection methods and histological subtypes. This outcome was comparable among patients from Asia or Africa, which could be due to the limited sample size and relatively poor literature quality. Although frozen and fresh tissues are better in the preservation of DNA quality than formalin‐fixed, paraffin‐embedded tissues, there were no differences between frozen and fresh tissues probably because of the small number of studies.

HPV‐associated oropharyngeal squamous cell carcinoma (OPSCC) was associated with worse overall survival outcomes, relative to non‐HPV‐associated OPSCC, even after adjusting for smoking status.^124^ Surprisingly, we found that HPV infection was significantly associated with BCa progression (RR = 1.73), especially BCa recurrence (RR = 1.87). This is highly attributed to the persistent existence of HPV E6 and E7 proteins in the bladder, which can inhibit tumor‐suppressing proteins and lead to carcinogenesis.^23^ This result shows the latent prognostic value of HPV infection for BCa.

Then, we investigated the causal relationship between HPV E7 protein levels and BCa risk. The meta‐analysis for different bladder cancer datasets showed that genetically elevated HPV E7 levels are causally associated with increased BCa risk for both HPV‐16 and HPV‐18, consistent with the findings from previous meta‐analyses of conventional observational studies after exclusion of latent bias caused by confounding factors. Although statistically significant, the OR values for HPV‐16 and HPV‐18 E7 proteins on BCa were close to 1 and dependent on E7 protein levels, therefore, this result should be carefully interpreted. Besides, clinical assessment of E7 protein levels is difficult and lacks practical significance. Apart from these limitations, findings from two‐sample MR analysis are in accordance with the result of this systematic review and meta‐analysis

This study has various limitations. First, due to the lack of original data, we did not investigate the role of HPV infection in different stages of bladder cancer. Second, most SCC cases were associated with *Schistosoma hematobium* infections, which could probably influence the outcomes of this study. Third, we only found a dataset for HPV E7 protein levels but did not find other instrumental variables for HPV infections. Fourth, there was a great heterogeneity in the characteristics of the study cohort. Fifth, detection methods for some studies were not sensitive enough and did not detect the specific HPV subtypes, which may have interfered with the final outcome. Finally, there were various limitations regarding our analysis of HPV and BCa progression. The number of studies included in this section was limited. Recurrence and death were not valid outcomes for prognosis, especially when the causes of death were not confirmed and specified and when the HPV persistence/clearance was unknown during BCa treatment/follow‐up. Several potential confounders during prognosis were unknown and were not controlled for in the analysis. Therefore, functional studies using cell lines and animals as well as clinical studies should be performed to confirm our findings.

## 5 CONCLUSION

HPV infection is significantly associated with increased risk and worse prognosis of bladder cancer. Therefore, HPV vaccination is necessary to prevent bladder cancer, especially among men. Future studies should investigate the association between HPV infection and bladder cancer using large‐scale sampling of various populations. Moreover, the mechanisms behind these phenomena should be elucidated.

## AUTHOR CONTRIBUTIONS

Jian‐Xuan Sun, Jin‐Zhou Xu, Chen‐Qian Liu, Qi‐Dong Xia, and Shao‐Gang Wang contributed to developing the main research question, carrying out the literature search, collecting the included studies’ information, and describing the results. Jian‐Xuan Sun, Jin‐Zhou Xu, and Chen‐Qian Liu performed the meta‐analysis and wrote the first draft of the manuscript. Xing‐Yu Zhong, Na Zeng, Hao‐Dong He, and Si‐Yang Ma contributed to developing the main research question and revised the manuscript. Ye An, Meng‐Yao Xu, Zheng Liu, and Jia Hu revised the manuscript. All authors contributed to the article and approved the submitted version. Jian‐Xuan Sun, Jin‐Zhou Xu, Chen‐Qian Liu, Qi‐Dong Xia, and Shao‐Gang Wang contributed equally to this study.

## CONFLICT OF INTEREST

The authors declare no conflict of interest.

## Supporting information

Supporting information.

Supporting information.

Supporting information.

Supporting information.

Supporting information.

Supporting information.

Supporting information.

Supporting information.

Supporting information.

Supporting information.

Supporting information.

Supporting information.

Supporting information.

Supporting information.

Supporting information.

Supporting information.

Supporting information.

Supporting information.

Supporting information.

Supporting information.

Supporting information.

Supporting information.

## Data Availability

The data that support the findings of this study are available from the corresponding author upon reasonable request. The original contributions presented in the study are included in the article/supplementary material, further inquiries can be directed to the corresponding author/s.
