# Supplementary material for: The association between human papillomavirus and bladder cancer: Evidence from meta‐analysis and two‐sample mendelian randomization
Source: J Med Virol. 2022 Oct 25;95(1):e28208. doi: 10.1002/jmv.28208 (PMC10092419; doi:10.1002/jmv.28208)

Variable	No. of studies	No. of cases	Proportion (%)	HPV prevalence (95%CI)	P
**Continent**					< 0.01
Europe	4	73	29.2	0.25 (0.03, 0.57)	
Oceania	1	4	2.2	0.02 (0.01, 0.05)	
North America	2	40	19.0	0.60 (0.00, 1.00)	
Asia	1	6	30.0	0.30 (0.12, 0.54)	
**Histological type**					< 0.01
TCC	6	93	68.6	0.17 (0.03, 0.38)	
SCC	1	6	10.0	0.30 (0.12, 0.54)	
**Study type**					< 0.01
Case-control study	3	50	44.2	0.59 (0.07, 1.00)	
Cross-sectional study	5	73	13.2	0.15 (0.01, 0.40)	
**Overall**	8	123	18.4	0.29 (0.06, 0.60)	

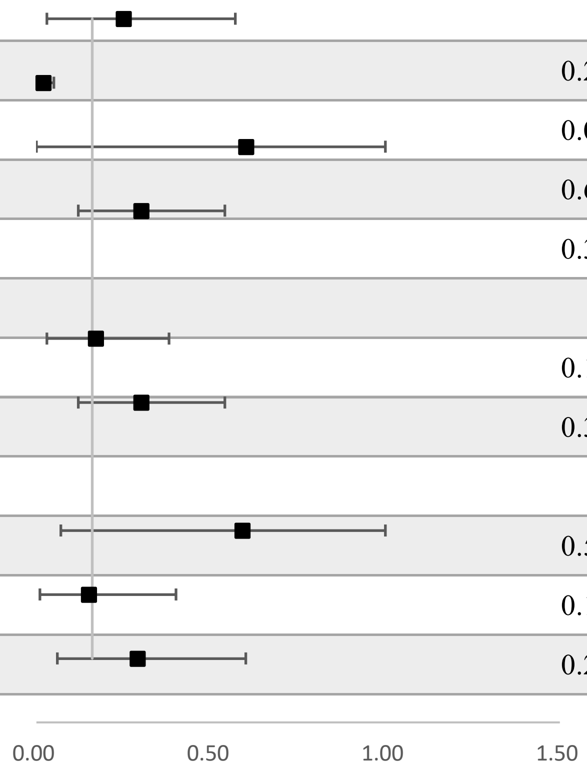

Supplement: Supplementary file 1 — Supporting information. [file JMV-95-0-s019.pdf]
