# Supplementary figures and images for: The association between human papillomavirus and bladder cancer: Evidence from meta‐analysis and two‐sample mendelian randomization
Source: J Med Virol. 2022 Oct 25;95(1):e28208. doi: 10.1002/jmv.28208 (PMC10092419; doi:10.1002/jmv.28208)

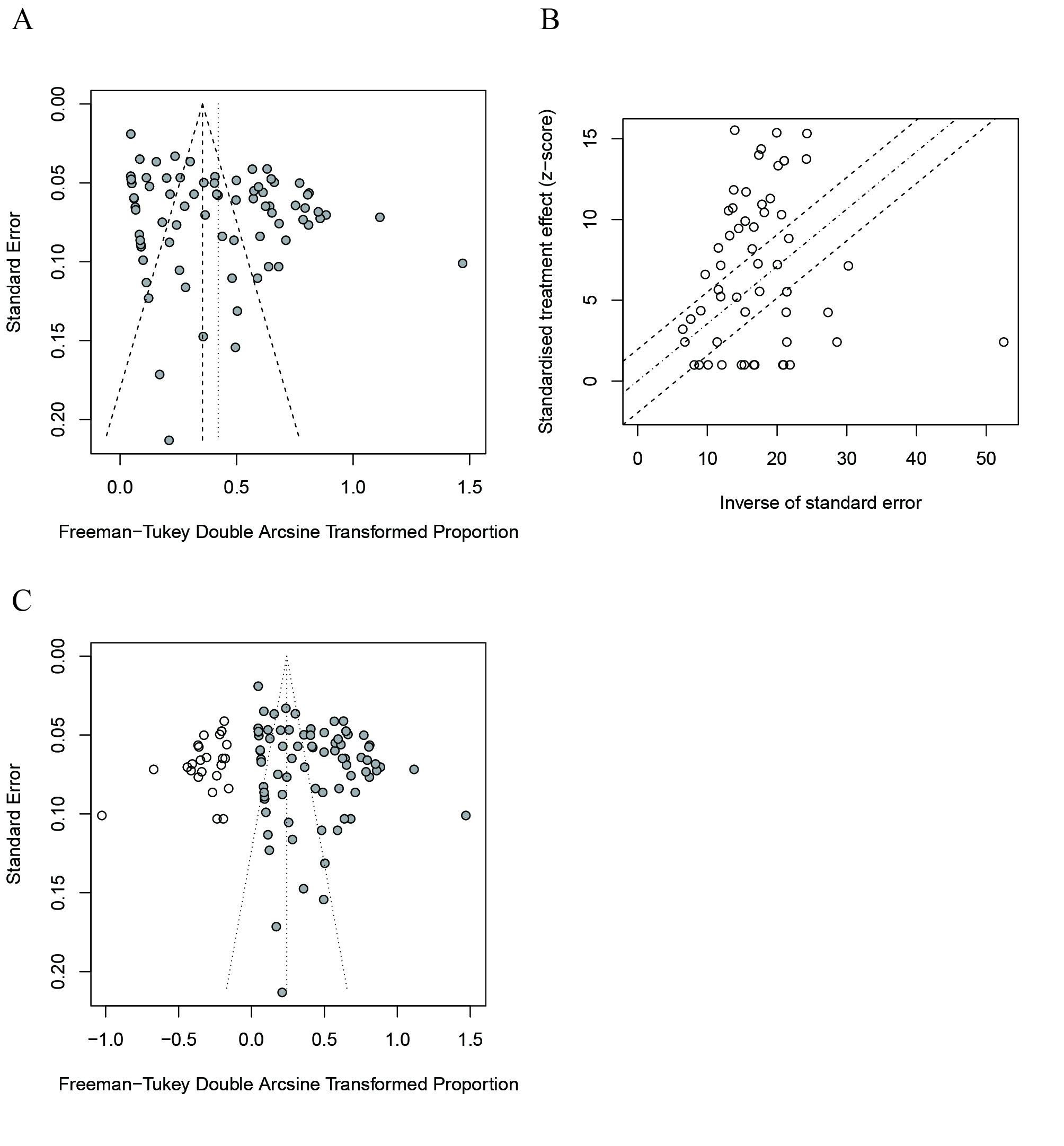

Supplement: Supplementary file 2 — Supporting information. [file JMV-95-0-s017.jpg]

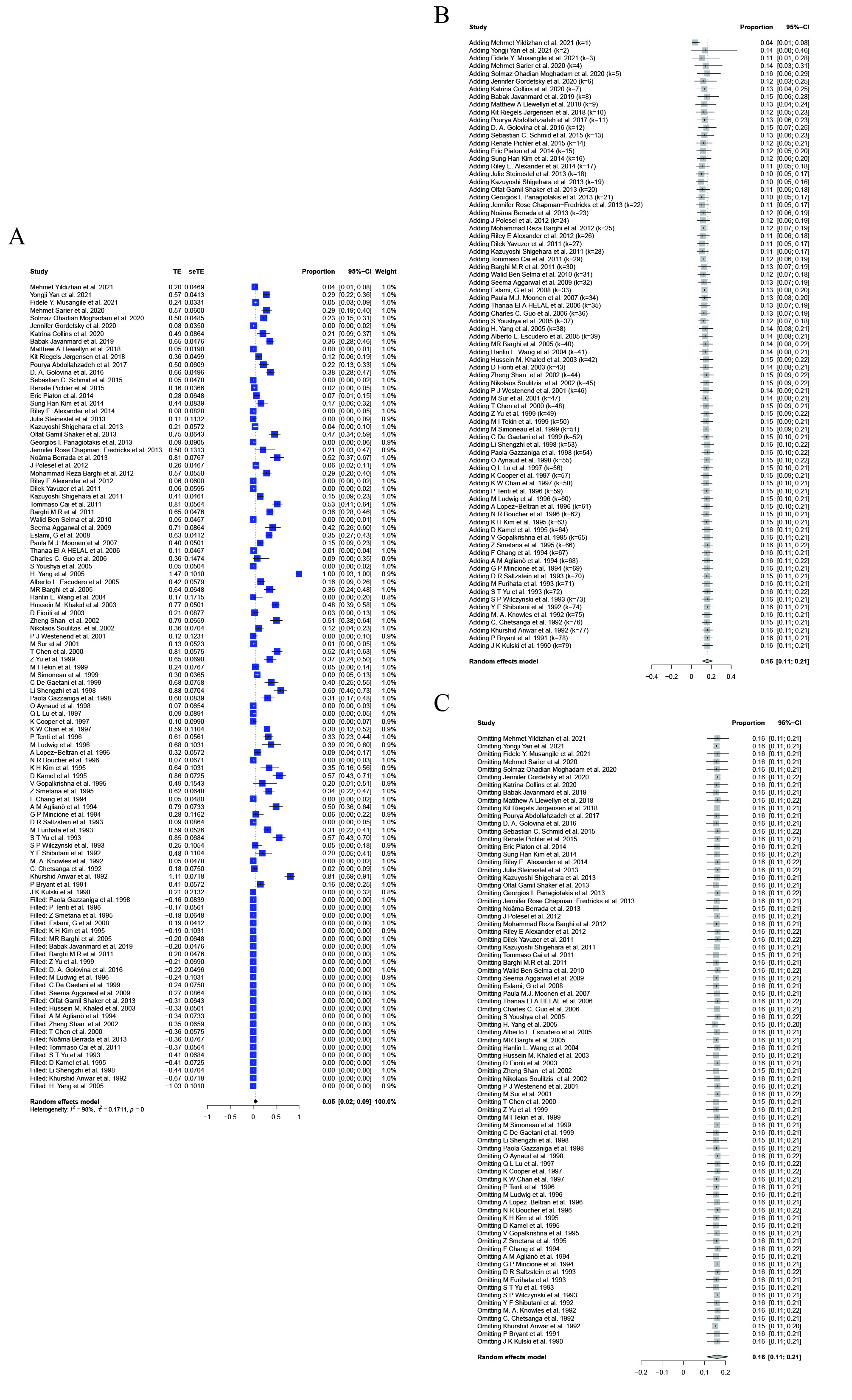

Supplement: Supplementary file 3 — Supporting information. [file JMV-95-0-s007.jpg]

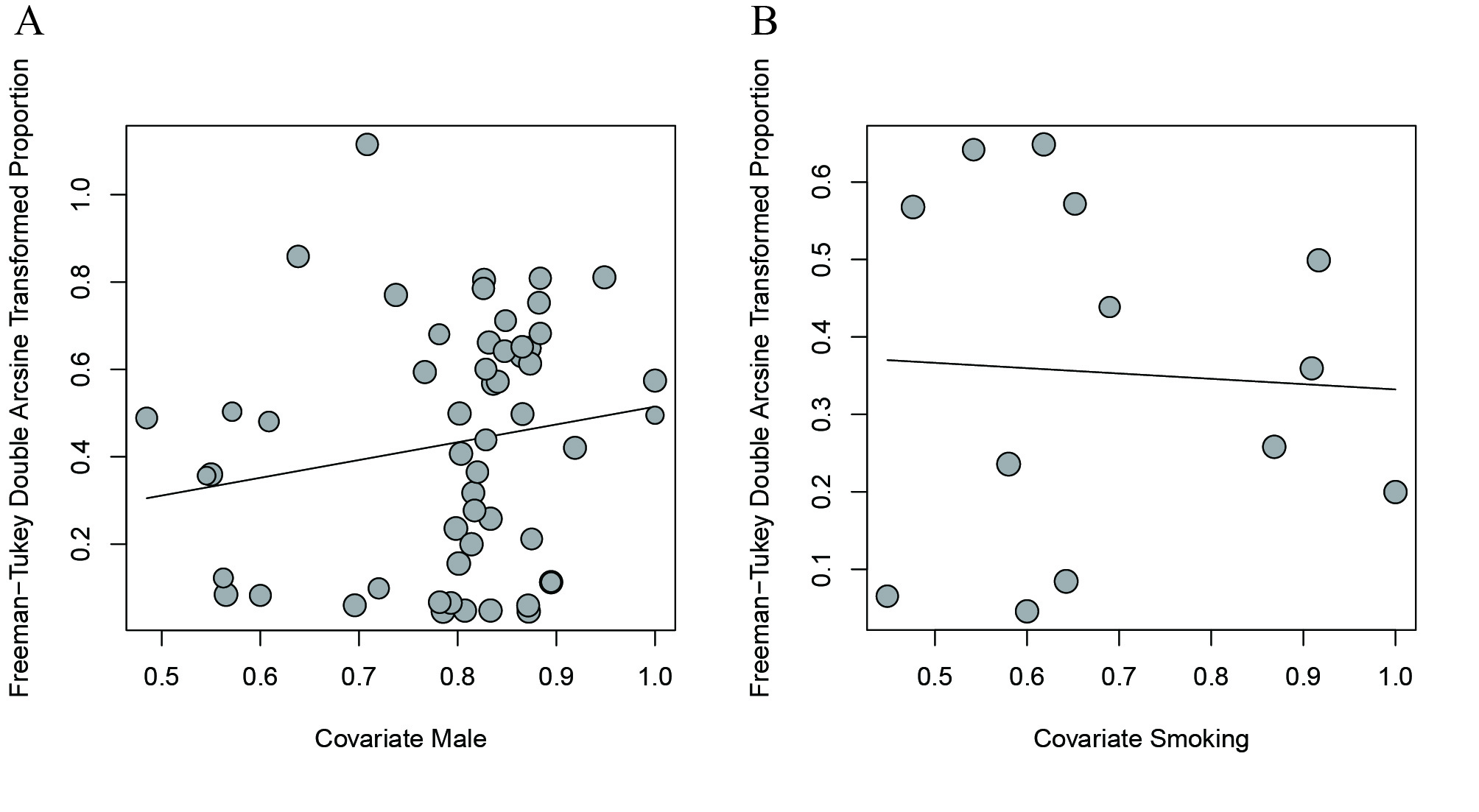

Supplement: Supplementary file 4 — Supporting information. [file JMV-95-0-s005.jpg]

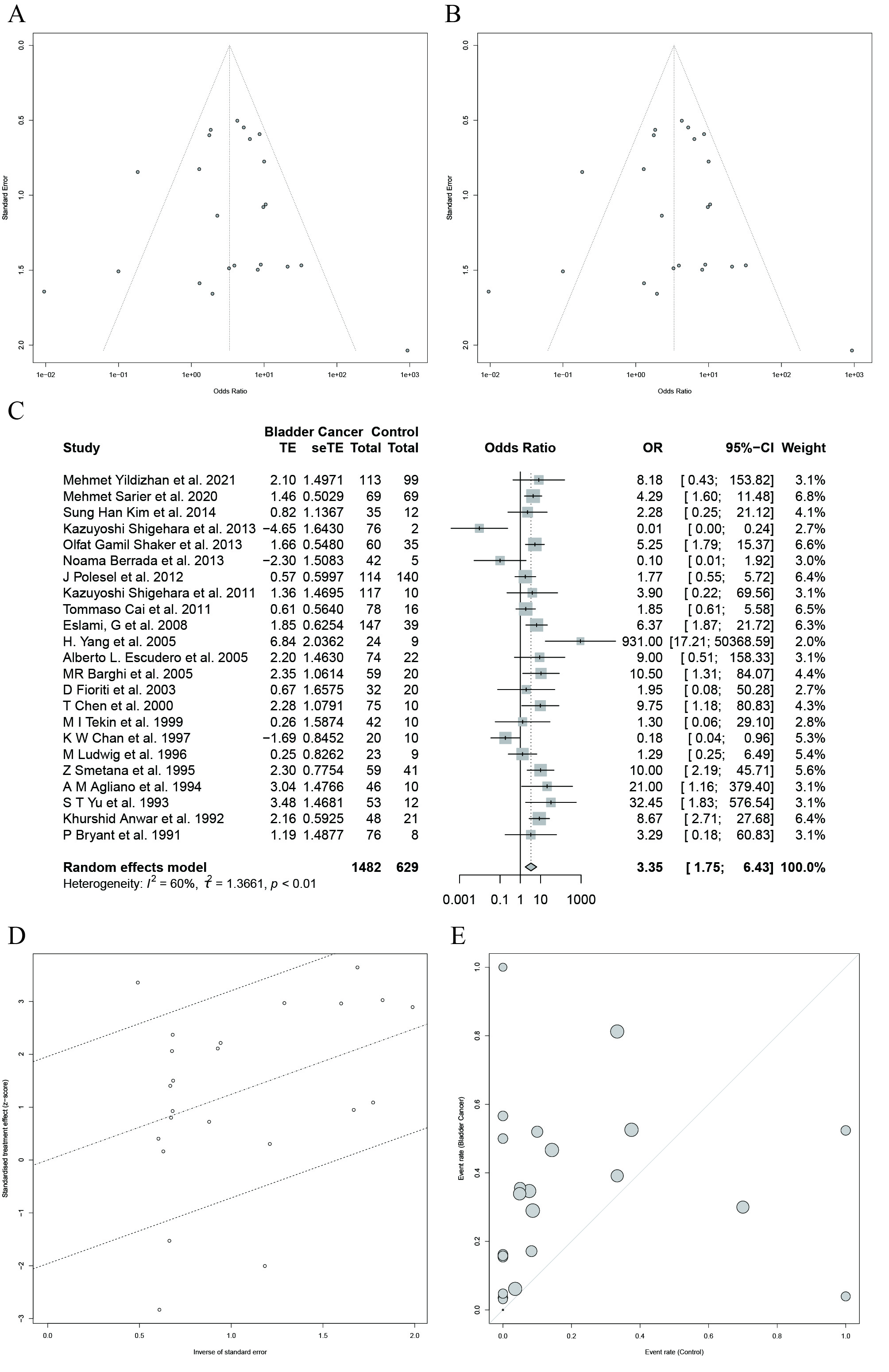

Supplement: Supplementary file 5 — Supporting information. [file JMV-95-0-s015.jpg]

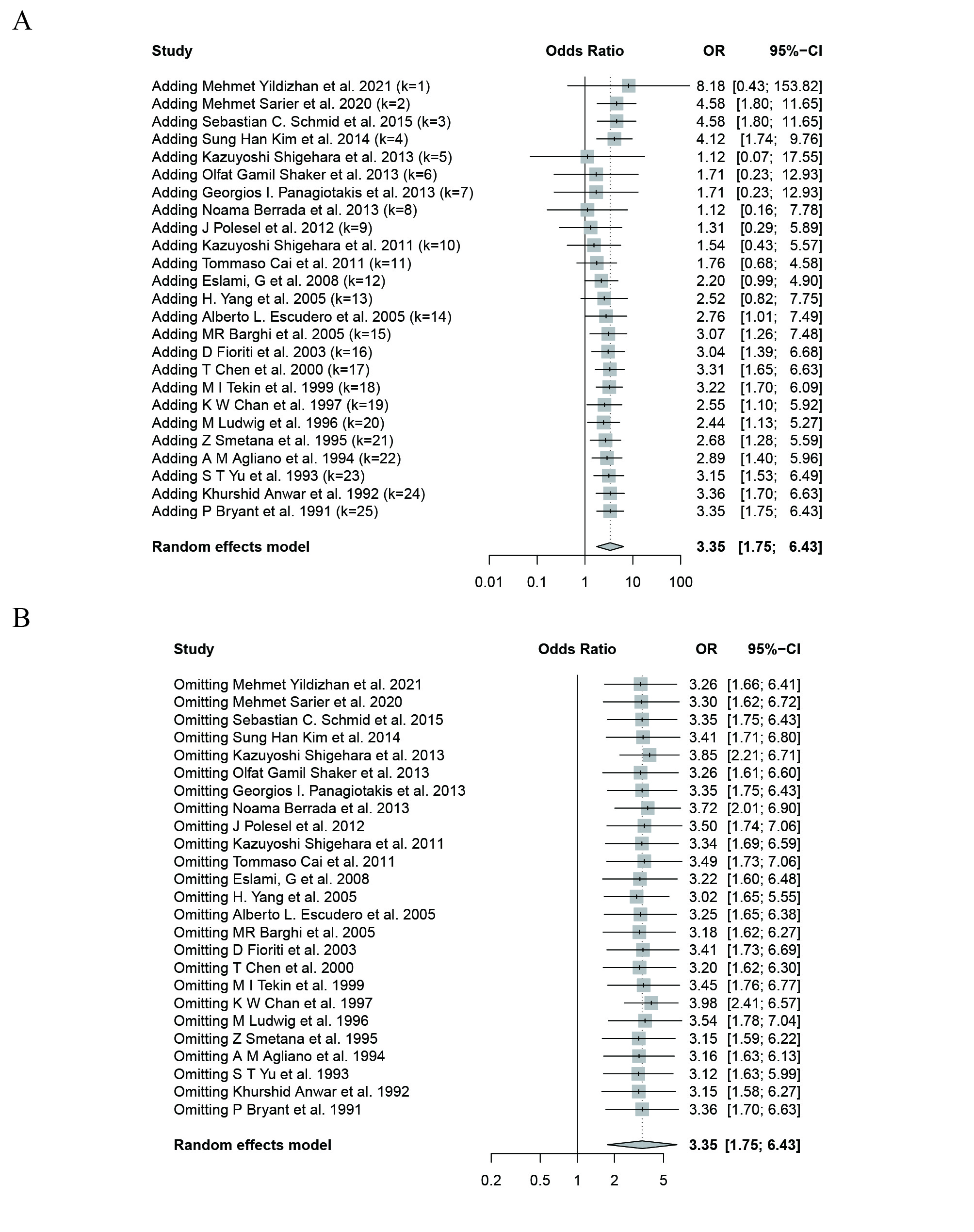

Supplement: Supplementary file 6 — Supporting information. [file JMV-95-0-s020.jpg]

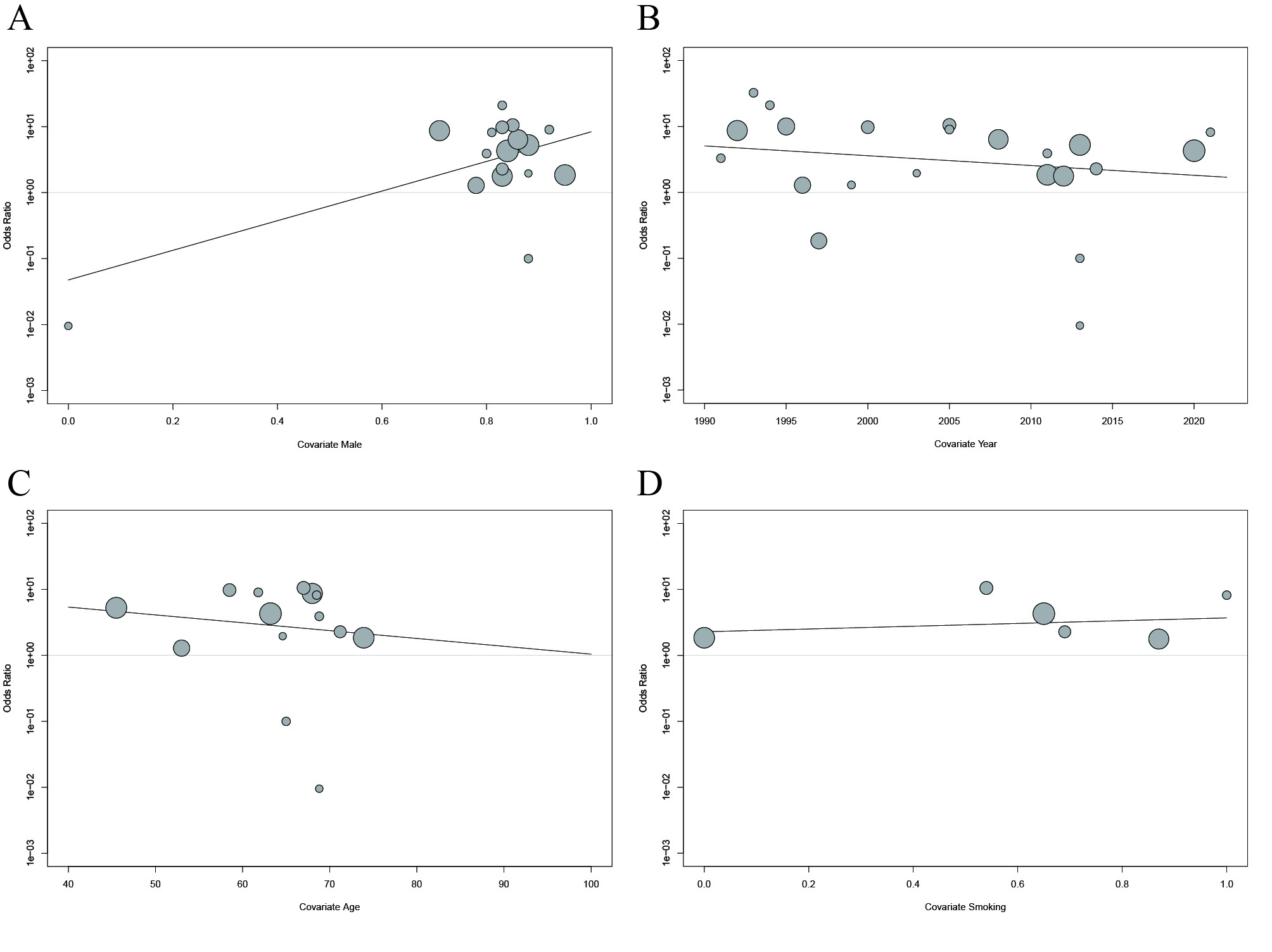

Supplement: Supplementary file 7 — Supporting information. [file JMV-95-0-s012.jpg]

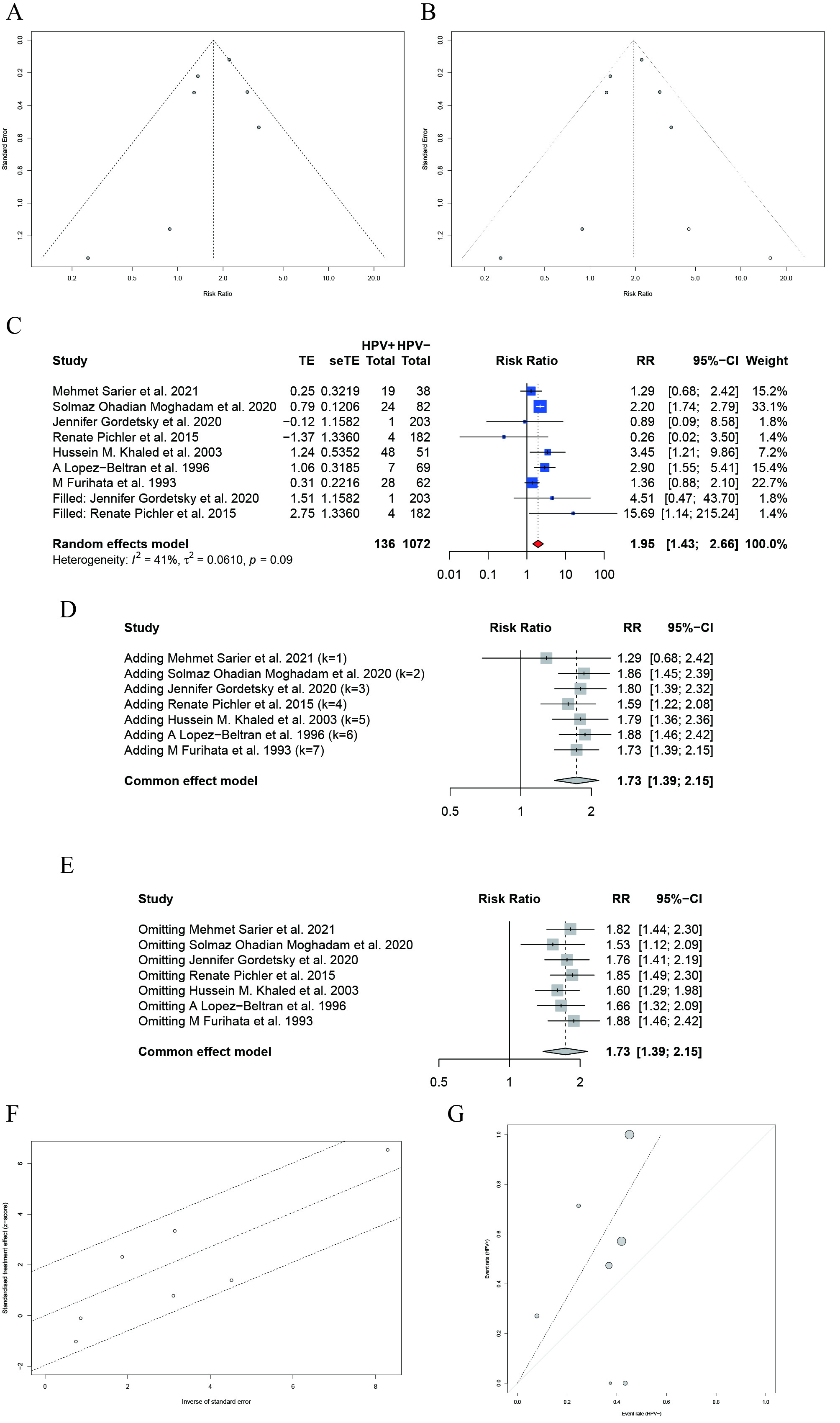

Supplement: Supplementary file 8 — Supporting information. [file JMV-95-0-s014.jpg]

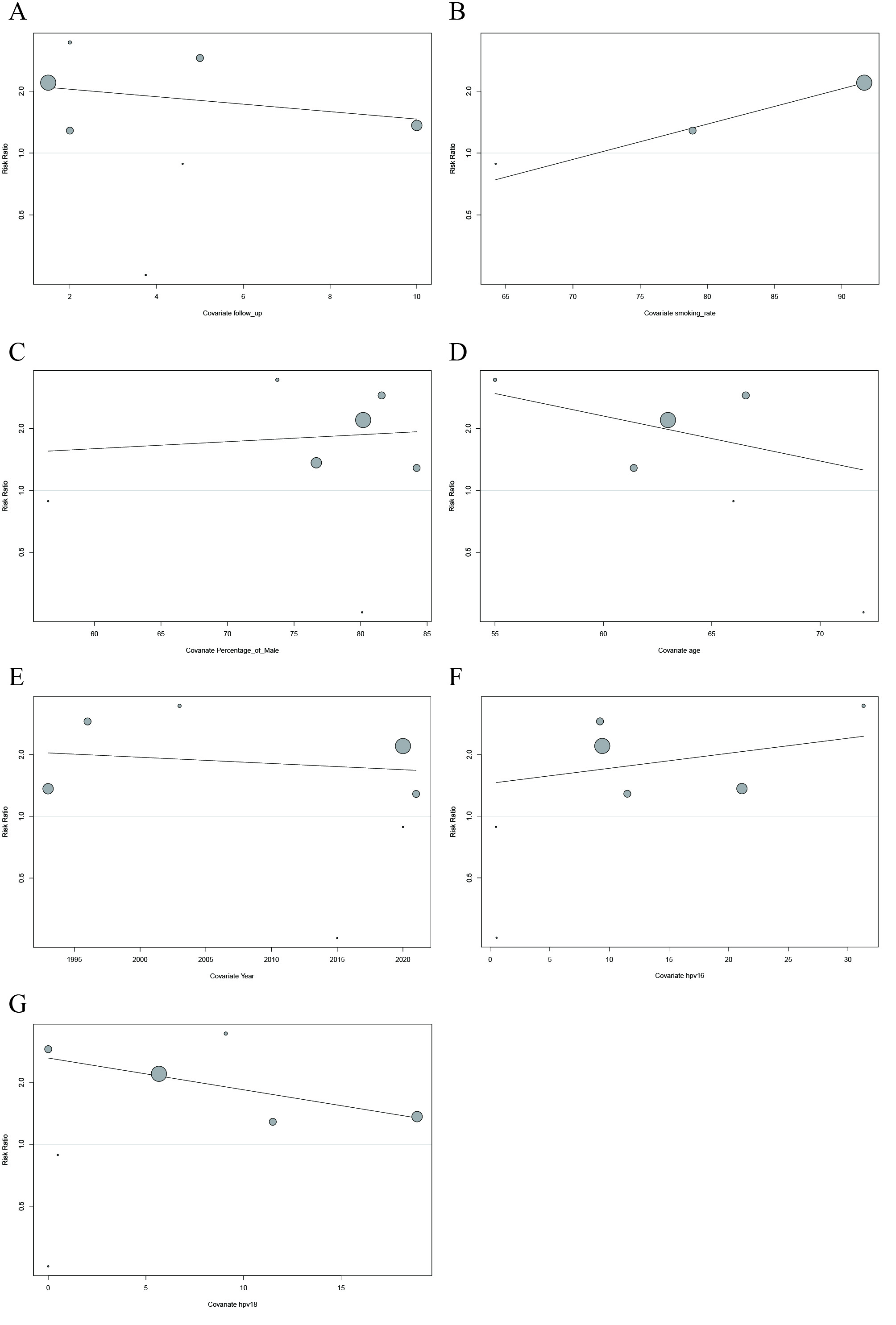

Supplement: Supplementary file 9 — Supporting information. [file JMV-95-0-s006.jpg]
