# Supplementary material for: The association between human papillomavirus and bladder cancer: Evidence from meta‐analysis and two‐sample mendelian randomization
Source: J Med Virol. 2022 Oct 25;95(1):e28208. doi: 10.1002/jmv.28208 (PMC10092419; doi:10.1002/jmv.28208)
Supplement: Supplementary file 10 — Supporting information. [file JMV-95-0-s009.docx]

**Table S1A: Details of Search Strategy to Retrieve the Studies using PubMed (Medline)**

**Date of Search: 5/21/2022**

| **#** | **Search Terms** | **Hits** |
| --- | --- | --- |
| #1 | Search: "Urinary Bladder Neoplasms"[Mesh] | 59,557 |
| #2 | Search: ((((((((((((((((((Urinary Bladder Neoplasms[Title/Abstract]) OR (Neoplasm, Urinary Bladder[Title/Abstract])) OR (Urinary Bladder Neoplasm[Title/Abstract])) OR (Bladder Tumors[Title/Abstract])) OR (Bladder Tumor[Title/Abstract])) OR (Tumor, Bladder[Title/Abstract])) OR (Tumors, Bladder[Title/Abstract])) OR (Neoplasms, Bladder[Title/Abstract])) OR (Bladder Neoplasms[Title/Abstract])) OR (Bladder Neoplasm[Title/Abstract])) OR (Neoplasm, Bladder[Title/Abstract])) OR (Urinary Bladder Cancer[Title/Abstract])) OR (Cancer, Urinary Bladder[Title/Abstract])) OR (Malignant Tumor of Urinary Bladder[Title/Abstract])) OR (Cancer of the Bladder[Title/Abstract])) OR (Bladder Cancer[Title/Abstract])) OR (Bladder Cancers[Title/Abstract])) OR (Cancer, Bladder[Title/Abstract])) OR (Cancer of Bladder[Title/Abstract]) | 47,742 |
| #3 | #1 OR #2 | 71,895 |
| #4 | Search: "Papillomavirus Infections"[Mesh] | 39,662 |
| #5 | Search: (((((((Papillomavirus Infections[Title/Abstract]) OR (Papillomavirus Infection[Title/Abstract])) OR (Human Papillomavirus Infection[Title/Abstract])) OR (Human Papillomavirus Infections[Title/Abstract])) OR (Papillomavirus Infection, Human[Title/Abstract])) OR (Papillomavirus Infections, Human[Title/Abstract])) OR (HPV Infection[Title/Abstract])) OR (HPV Infections[Title/Abstract]) | 16,240 |
| #6 | Search: ("Papillomavirus Infections"[Mesh]) OR ((((((((Papillomavirus Infections[Title/Abstract]) OR (Papillomavirus Infection[Title/Abstract])) OR (Human Papillomavirus Infection[Title/Abstract])) OR (Human Papillomavirus Infections[Title/Abstract])) OR (Papillomavirus Infection, Human[Title/Abstract])) OR (Papillomavirus Infections, Human[Title/Abstract])) OR (HPV Infection[Title/Abstract])) OR (HPV Infections[Title/Abstract])) | 44,942 |
| **#7** | **#3 and #6** | **245** |

**Table S1B: Details of Search Strategy to Retrieve the Studies using Embase**

**Date of Search: 5/21/2022**

| **#** | **Search Terms** | **Hits** |
| --- | --- | --- |
| #1 | (bladder cancer or urinary bladder cancer or urine bladder cancer or vesical cancer or bladder tumor or urinary tract cancer or bladder carcinogenesis or bladder carcinoma or bladder metastasis or muscle invasive bladder cancer or non muscle invasive bladder cancer).ab,kw,ti. | 66,785 |
| #2 | (papillomavirus infection or papillomavirus infections or Human papillomavirus infection).ab,kw,ti. | 5,496 |
| **#3** | **#1 AND #2** | **24** |

**Table S1C: Details of Search Strategy to Retrieve the Studies using Cochrane**

**Date of Search: 5/21/2022**

| **#** | **Search Terms** | **Hits** |
| --- | --- | --- |
| #1 | (Neoplasm, Urinary Bladder):ti,ab,kw or (Urinary Bladder Neoplasm):ti,ab,kw or (Bladder Tumors):ti,ab,kw or (Bladder Tumor):ti,ab,kw or (Tumor, Bladder):ti,ab,kw or (Tumors, Bladder):ti,ab,kw or (Neoplasms, Bladder):ti,ab,kw or (Bladder Neoplasms):ti,ab,kw or (Bladder Neoplasm):ti,ab,kw or (Neoplasm, Bladder):ti,ab,kw or (Urinary Bladder Cancer):ti,ab,kw or (Cancer, Urinary Bladder):ti,ab,kw or (Malignant Tumor of Urinary Bladder):ti,ab,kw or (Cancer of the Bladder):ti,ab,kw or (Bladder Cancer):ti,ab,kw or (Bladder Cancers):ti,ab,kw or (Cancer, Bladder):ti,ab,kw or (Cancer of Bladder):ti,ab,kw | 5,753 |
| #2 | (Papillomavirus Infection):ti,ab,kw or (Human Papillomavirus Infection):ti,ab,kw or (Human Papillomavirus Infections):ti,ab,kw or (Papillomavirus Infection, Human):ti,ab,kw or (Papillomavirus Infections, Human):ti,ab,kw or (HPV Infection):ti,ab,kw or (HPV Infections):ti,ab,kw | 1,872 |
| **#3** | **#1 AND #2** | **2** |

**Table S1D: Details of Search Strategy to Retrieve the Studies using Web of Science**

**Date of Search: 5/21/2022**

| **#** | **Search Terms** | **Hits** |
| --- | --- | --- |
| #1 | TS = "Neoplasm, Urinary Bladder" OR "Urinary Bladder Neoplasm" OR "Bladder Tumors" OR "Bladder Tumor" OR "Tumor, Bladder" OR "Tumors, Bladder" OR "Neoplasms, Bladder" OR "Bladder Neoplasms" OR "Bladder Neoplasm" OR "Neoplasm, Bladder" OR "Urinary Bladder Cancer" OR "Cancer, Urinary Bladder" OR "Malignant Tumor of Urinary Bladder" OR "Cancer of the Bladder" OR "Bladder Cancer" OR "Bladder Cancers" OR "Cancer, Bladder" OR "Cancer of Bladder" | 131,881 |
| #2 | TS = "Papillomavirus Infection" OR "Human Papillomavirus Infection" OR "Human Papillomavirus Infections" OR "Papillomavirus Infection, Human" OR "Papillomavirus Infections, Human" OR "HPV Infection" OR "HPV Infections" | 35,971 |
| **#3** | **#1 AND #2** | **269** |
