# Supplementary material for: The association between human papillomavirus and bladder cancer: Evidence from meta‐analysis and two‐sample mendelian randomization
Source: J Med Virol. 2022 Oct 25;95(1):e28208. doi: 10.1002/jmv.28208 (PMC10092419; doi:10.1002/jmv.28208)
Supplement: Supplementary file 11 — Supporting information. [file JMV-95-0-s004.docx]

**Table S2 The prevalence of HPV types in bladder cancer cases of the included studies in this systematic review and meta-analysis.**

| **Study (Year)** | **HPV11 (%)** | | **HPV16 (%)** | **HPV18 (%)** | **HPV26 (%)** | **HPV31 (%)** | **HPV33 (%)** | **HPV35 (%)** | **HPV39 (%)** | **HPV40 (%)** | **HPV43 (%)** | **HPV45 (%)** | **HPV51 (%)** | **HPV52 (%)** | **HPV53 (%)** | **HPV56 (%)** | **HPV58 (%)** | **HPV66 (%)** | **HPV68 (%)** | **HPV70 (%)** | **HPV73 (%)** | **HPV82 (%)** | **HPV84 (%)** |
| --- | --- | --- | --- | --- | --- | --- | --- | --- | --- | --- | --- | --- | --- | --- | --- | --- | --- | --- | --- | --- | --- | --- | --- |
| Mehmet Yildizhan et al. (2021) |  | |  |  |  |  |  |  |  |  |  |  |  |  | 1 (0.88) |  |  | 1 (0.88) |  |  |  |  | 1 (0.88) |
| Yongji Yan et al. (2021) |  | | 9 (6.16) | 36 (24.66) |  |  | 17 (11.64) |  | 2 (1.37) |  |  |  |  |  |  |  |  |  |  |  |  |  |  |
| Mehmet Sarier et al. (2021) |  | | 3 (11.5) | 3 (11.5) | 1 (3.9) |  |  |  | 3 (11.5) |  |  | 1 (3.9) | 2 (7.7) |  | 3 (11.5) | 1 (3.9%) |  | 2 (7.7%) | 2 (7.7%) |  |  | 2 (7.7%) |  |
| Fidele Y. Musangile et al. (2021) |  | | 1 (0.44) | 1 (0.44) |  |  |  |  |  |  |  |  |  | 1 (0.44) |  |  |  |  |  |  |  |  |  |
| Mehmet Sarier et al. (2020) |  | | 3 (11.1) | 3 (11.1) | 1 (3.7) |  |  |  | 3 (11.1) |  |  | 1 (3.7) | 2 (7.4) |  | 3 (11.1) | 1 (3.7) |  | 2 (7.4) | 2 (7.4) |  |  | 2 (7.4) |  |
| Solmaz Ohadian Moghadam et al. (2020) | 6 (5.67) | | 10 (9.4) | 6 (5.67) |  |  |  |  |  |  |  |  |  |  |  |  |  |  |  |  |  |  |  |
| Babak Javanmard et al. (2019) |  | | 25 (22.73) | 27 (24.55) |  |  |  |  |  |  |  |  |  |  |  |  |  |  |  |  |  |  |  |
| Matthew A Llewellyn et al. (2018) |  | | 1 (0.15) |  |  |  |  |  |  |  |  |  |  |  |  |  |  |  |  |  |  |  |  |
| Kit Riegels Jørgensen et al. (2018) |  | | 3 (3) |  |  |  |  |  |  |  |  |  | 1 (1) |  |  |  |  |  |  |  |  |  |  |
| D. A. Golovina et al. (2016) |  | | 38 (37.62) |  |  |  |  |  |  |  |  |  |  |  |  |  |  |  |  |  |  |  |  |
| Sebastian C. Schmid et al. (2015) |  | |  |  |  |  |  |  |  |  |  |  |  |  |  |  |  |  |  |  |  |  |  |
| Renate Pichler et al. (2015) |  | | 1 (0.54) |  |  |  |  |  |  |  |  |  |  |  |  |  |  |  |  |  |  |  |  |
| Eric Piaton et al. (2014) |  | | 1 (1.69) |  |  | 1 (1.69) |  |  |  |  |  |  |  |  |  |  |  |  |  | 1 (1.69) |  |  | 1 (1.69) |
| Sung Han Kim et al. (2014) |  | |  | 5 (14.29) |  |  |  | 1 (2.86) |  |  |  |  |  |  |  |  |  |  |  |  |  |  |  |
| Kazuyoshi Shigehara et al. (2013) |  | | 2 (2.63) |  |  |  |  |  |  |  |  |  |  | 1 (1.32) |  |  |  |  |  |  |  |  |  |
| Olfat Gamil Shaker et al. (2013) | 23 (38.33) | | |  |  |  |  |  |  |  |  |  |  |  |  |  |  |  |  |  |  |  |  |
| Jennifer Rose Chapman-Fredricks et al. (2013) |  | | 2 (14.29) |  |  |  |  | 1 (7.14) |  |  |  |  |  |  |  |  |  |  |  |  |  |  |  |
| Noâma Berrada et al. (2013) |  | | 22 (52.38) |  |  | 2 (4.76) |  |  |  |  |  |  |  |  |  |  |  |  |  |  |  |  |  |
| J Polesel et al. (2012) |  | |  |  |  | 1 (0.88) |  | 1 (0.88) |  |  |  | 1 (0.88) |  |  |  | 2 (1.75) | 1 (0.88) |  |  | 1 (0.88) |  |  |  |
| Mohammad Reza Barghi et al. (2012) |  | | 3 (3.66) | 9 (10.98) |  |  |  |  |  |  |  |  |  |  |  |  |  |  |  |  |  |  |  |
| Kazuyoshi Shigehara et al. (2011) |  | | 6 (5.13) | 4 (3.42) |  | 1 (0.85) | 3 (2.56) |  |  |  |  |  |  | 1 (0.85) |  | 1 (0.85) | 1 (0.85) |  |  |  |  |  |  |
| Tommaso Cai et al. (2011) | 3 (3.85) | | 4 (5.13) | 6 (7.69) |  | 3 (3.85) | 2 (2.56) |  |  | 1 (1.28) | 2 (1.28) | 5 (6.41) | 2 (2.56) |  |  |  | 3 (3.85) | 4 (5.13) |  |  | 2 (2.56) |  |  |
| Barghi M.R et al. (2011) |  | | 25 (22.73) | 27 (24.55) |  |  |  |  |  |  |  |  |  |  |  |  |  |  |  |  |  |  |  |
| Seema Aggarwal et al. (2009) |  | | 14 (42.42) | 8 (24.24) |  |  |  |  |  |  |  |  |  |  |  |  |  |  |  |  |  |  |  |
| Paula M.J. Moonen et al. (2007) | 2 (2.02) | | 2 (2.02) | 3 (3.03) |  | 1 (1.01) | 1 (1.01) |  | 1 (1.01) | 1 (1.01) |  |  |  | 1 (1.01) |  |  |  |  |  |  |  |  |  |
| Thanaa El A HELAL et al. (2006) |  | | 1 (0.88) | |  |  |  |  |  |  |  |  |  |  |  |  |  |  |  |  |  |  |  |
| H. Yang et al. (2005) |  | | 24 (100) |  |  |  |  |  |  |  |  |  |  |  |  |  |  |  |  |  |  |  |  |
| Alberto L. Escudero et al. (2005) |  | | 12 (16.22) |  |  |  |  |  |  |  |  |  |  |  |  |  |  |  |  |  |  |  |  |
| MR Barghi et al. (2005) |  | |  | 14 (23.73) |  |  | 2 (3.39) |  |  |  |  |  |  |  |  |  |  |  |  |  |  |  |  |
| Hussein M. Khaled et al. (2003) | 31 (31.31) | 9 (9.09) | |  |  |  |  |  |  |  |  |  |  |  |  |  |  |  |  |  |  |  |  |
| Zheng Shan et al. (2002) |  | |  | 29 (50.88) |  |  |  |  |  |  |  |  |  |  |  |  |  |  |  |  |  |  |  |
| Nikolaos Soulitzis et al. (2002) |  | |  | 6 (12) |  |  |  |  |  |  |  |  |  |  |  |  |  |  |  |  |  |  |  |
| T Chen et al. (2000) | 4 (5.33) | | 25 (33.33) | 5 (6.67) |  |  |  |  |  |  |  |  |  |  |  |  |  |  |  |  |  |  |  |
| Z Yu et al. (1999) |  | | 8 (15.38) | 13 (25) |  |  |  |  |  |  |  |  |  |  |  |  |  |  |  |  |  |  |  |
| M I Tekin et al. (1999) |  | | 2 (4.76) |  |  |  |  |  |  |  |  |  |  |  |  |  |  |  |  |  |  |  |  |
| M Simoneau et al. (1999) | 1 (0.53) | | 9 (4.81) | 4 (2.14) |  |  |  |  |  |  |  |  |  |  |  |  |  |  |  |  |  |  |  |
| C De Gaetani et al. (1999) | 6 (13.95) | | |  | 10 (23.26) | | |  |  |  |  |  |  |  |  |  |  |  |  |  |  |  |  |
| Li Shengzhi et al. (1998) |  | | 5 (10) | |  |  |  |  |  |  |  |  |  |  |  |  |  |  |  |  |  |  |  |
| Paola Gazzaniga et al. (1998) |  | | 6 (17.14) | 5 (14.29) |  |  |  |  |  |  |  |  |  |  |  |  |  |  |  |  |  |  |  |
| K W Chan et al. (1997) |  | |  | 6 (30) |  |  |  |  |  |  |  |  |  |  |  |  |  |  |  |  |  |  |  |
| P Tenti et al. (1996) |  | | 23 (29.11) | 10 (12.66) |  |  |  |  |  |  |  |  |  |  |  |  |  |  |  |  |  |  |  |
| M Ludwig et al. (1996) |  | | 2 (8.70) | |  |  |  |  |  |  |  |  |  |  |  |  |  |  |  |  |  |  |  |
| A Lopez-Beltran et al. (1996) |  | | 7 (9.21) |  |  |  |  |  |  |  |  |  |  |  |  |  |  |  |  |  |  |  |  |
| N R Boucher et al. (1996) |  | | 4 (17.39) | 8 (34.78) |  |  |  |  |  |  |  |  |  |  |  |  |  |  |  |  |  |  |  |
| K H Kim et al. (1995) |  | |  |  |  |  |  |  |  |  |  |  |  |  |  |  |  |  |  |  |  |  |  |
| D Kamel et al. (1995) | 10 (21.28) | | 10 (21.28) | 16 (34.04) |  | 19 (40.43) | 13 (27.66) |  |  |  |  |  |  |  |  |  |  |  |  |  |  |  |  |
| V Gopalkrishna et al. (1995) |  | | 2 (20) |  |  |  |  |  |  |  |  |  |  |  |  |  |  |  |  |  |  |  |  |
| Z Smetana et al. (1995) | 8 (13.56) | | |  |  |  |  |  |  |  |  |  |  |  |  |  |  |  |  |  |  |  |  |
| A M Aglianò et al. (1994) |  | | 18 (39.13) | 12 (26.09) |  |  |  |  |  |  |  |  |  |  |  |  |  |  |  |  |  |  |  |
| M Furihata et al. (1993) |  | | 19 (21.11) | 17 (18.89) |  |  | 16 (17.78) |  |  |  |  |  |  |  |  |  |  |  |  |  |  |  |  |
| S T Yu et al. (1993) |  | | 28 (52.83) | 2 (3.77) |  |  |  |  |  |  |  |  |  |  |  |  |  |  |  |  |  |  |  |
| S P Wilczynski et al. (1993) |  | |  |  |  |  |  |  |  |  |  |  |  |  |  |  |  |  |  |  |  |  |  |
| Y F Shibutani et al. (1992) | 1 (5) | | |  | 1 (5) | |  |  |  |  |  |  |  |  |  |  |  |  |  |  |  |  |  |
| M. A. Knowles et al. (1992) |  | |  |  |  |  |  |  |  |  |  |  |  |  |  |  |  |  |  |  |  |  |  |
| C. Chetsanga et al. (1992) |  | | 1 (2.27) |  |  |  |  |  |  |  |  |  |  |  |  |  |  |  |  |  |  |  |  |
| Khurshid Anwar et al. (1992) |  | | 13 (27.08) | 18 (37.5) |  |  | 14 (29.17) |  |  |  |  |  |  |  |  |  |  |  |  |  |  |  |  |
| P Bryant et al. (1991) |  | | 12 (15.79) | |  |  |  |  |  |  |  |  |  |  |  |  |  |  |  |  |  |  |  |

HPV, human papilloma virus
