# Supplementary material for: The association between human papillomavirus and bladder cancer: Evidence from meta‐analysis and two‐sample mendelian randomization
Source: J Med Virol. 2022 Oct 25;95(1):e28208. doi: 10.1002/jmv.28208 (PMC10092419; doi:10.1002/jmv.28208)
Supplement: Supplementary file 12 — Supporting information. [file JMV-95-0-s022.docx]

**Table S3 Agency for Healthcare Research and Quality (AHRQ) evaluation criteria for assessing the quality of included cross-sectional studies.**

| **Author (Year)** | **Define the source of information (survey, record review)** | **List inclusion and exclusion criteria for exposed and unexposed subjects (cases and controls) or refer to previous publications** | **Indicate time period used for identifying patients** | **Indicate whether or not subjects were consectutive if not population-based** | **Indicate if evaluators of subjective components of study were masked to other aspects of the status of the participants** | **Describle any assessments undertaken for quality assurance purposes (e.g., test/retest of primary outcome measurements)** | **Explain any patient exclusions from analysis** | **Describle how confounding was assessed and/or controlled** | **If applicable, explain how missing data were handled in the analysis** | **Summarize patient response rates and completeness of data collection** | **Clarify what follow-up, if any, was expected and the percentage of patients for which incomplete data of follow-up was obtained** | **Total** |
| --- | --- | --- | --- | --- | --- | --- | --- | --- | --- | --- | --- | --- |
| Mehmet Yıldızhan et al. (2021) | 1 | 1 | 1 | 1 | 1 | 1 | 1 | 0 | 0 | 0 | 0 | 7 |
| Yongji Yan et al. (2021) | 1 | 0 | 1 | 1 | 1 | 1 | 0 | 0 | 0 | 1 | 0 | 6 |
| Mehmet Sarier et al. (2021) | 1 | 1 | 1 | 1 | 1 | 1 | 0 | 0 | 0 | 0 | 1 | 7 |
| Fidele Y. Musangile et al. (2021) | 1 | 1 | 1 | 1 | 1 | 1 | 0 | 0 | 0 | 1 | 0 | 7 |
| Solmaz Ohadian Moghadam et al. (2020) | 1 | 0 | 0 | 0 | 1 | 1 | 0 | 1 | 0 | 0 | 1 | 5 |
| Jennifer Gordetsky et al. (2020) | 1 | 1 | 1 | 0 | 1 | 1 | 1 | 0 | 1 | 1 | 1 | 9 |
| Katrina Collins et al. (2020) | 1 | 1 | 1 | 1 | 1 | 1 | 1 | 0 | 0 | 1 | 1 | 9 |
| Babak Javanmard et al. (2019) | 1 | 1 | 1 | 1 | 1 | 0 | 0 | 0 | 0 | 0 | 0 | 5 |
| Matthew A Llewellyn et al. (2018) | 1 | 0 | 1 | 1 | 1 | 1 | 0 | 0 | 0 | 1 | 0 | 6 |
| Kit Riegels Jørgensen et al. (2018) | 1 | 1 | 1 | 1 | 1 | 0 | 0 | 0 | 0 | 1 | 0 | 6 |
| Pourya Abdollahzadeh et al. (2017) | 1 | 0 | 1 | 1 | 1 | 0 | 0 | 0 | 0 | 0 | 0 | 4 |
| D. A. Golovina et al. (2016) | 1 | 1 | 0 | 1 | 1 | 0 | 0 | 0 | 0 | 0 | 0 | 4 |
| Renate Pichler et al. (2015) | 1 | 1 | 1 | 1 | 1 | 1 | 0 | 0 | 0 | 1 | 0 | 7 |
| Eric Piaton et al. (2014) | 1 | 1 | 0 | 1 | 1 | 1· | 0 | 0 | 0 | 1 | 0 | 6 |
| Riley E. Alexander et al. (2014) | 1 | 1 | 0 | 0 | 1 | 1 | 1 | 0 | 0 | 1 | 0 | 6 |
| Jennifer Rose Chapman-Fredricks et al. (2013) | 1 | 1 | 0 | 1 | 1 | 1 | 0 | 0 | 0 | 1 | 0 | 6 |
| Mohammad Reza Barghi et al. (2012) | 1 | 1 | 1 | 1 | 1 | 1 | 1 | 0 | 0 | 1 | 0 | 8 |
| Riley E Alexander et al. (2012) | 1 | 1 | 1 | 1 | 1 | 0 | 1 | 0 | 0 | 1 | 0 | 7 |
| Dilek Yavuzer et al. (2011) | 1 | 1 | 0 | 1 | 1 | 0 | 0 | 0 | 0 | 0 | 0 | 4 |
| Walid Ben Selma et al. (2010) | 1 | 1 | 1 | 1 | 1 | 0 | 0 | 0 | 0 | 0 | 0 | 5 |
| Seema Aggarwal et al. (2009) | 1 | 1 | 0 | 1 | 1 | 0 | 0 | 0 | 0 | 0 | 0 | 4 |
| Paula M.J. Moonen et al. (2007) | 1 | 1 | 0 | 1 | 1 | 0 | 1 | 0 | 0 | 0 | 0 | 5 |
| Thanaa El A HELAL et al. (2006) | 1 | 1 | 0 | 1 | 1 | 0 | 0 | 0 | 0 | 0 | 0 | 4 |
| Charles C. Guo et al. (2006) | 1 | 1 | 0 | 1 | 1 | 0 | 0 | 1 | 0 | 0 | 1 | 6 |
| S Youshya et al. (2005) | 1 | 1 | 0 | 1 | 1 | 1 | 0 | 0 | 0 | 0 | 0 | 5 |
| Hanlin L. Wang et al.（2004） | 1 | 1 | 1 | 1 | 1 | 1 | 1 | 0 | 0 | 0 | 0 | 7 |
| Hussein M. Khaled et al.（2003） | 1 | 1 | 1 | 1 | 1 | 1 | 0 | 1 | 0 | 1 | 1 | 9 |
| Nikolaos Soulitzis et al. (2002) | 1 | 1 | 1 | 1 | 1 | 0 | 0 | 0 | 0 | 0 | 0 | 5 |
| P J Westenend et al. (2001) | 1 | 1 | 0 | 0 | 1 | 1 | 1 | 0 | 0 | 0 | 0 | 5 |
| M Sur et al.（2001） | 1 | 1 | 0 | 1 | 1 | 1 | 0 | 0 | 0 | 0 | 0 | 5 |
| Z Yu et al.（1999） | 1 | 1 | 1 | 1 | 1 | 1 | 0 | 0 | 0 | 0 | 0 | 6 |
| M Simoneau et al. (1999) | 1 | 1 | 1 | 1 | 1 | 1 | 0 | 0 | 0 | 0 | 0 | 6 |
| C De Gaetani et al.（1999） | 1 | 1 | 1 | 1 | 1 | 1 | 0 | 0 | 0 | 0 | 0 | 6 |
| Li Shengzhi et al.（1998） | 1 | 1 | 0 | 0 | 1 | 1 | 0 | 0 | 0 | 0 | 0 | 4 |
| Paola Gazzaniga et al., 1998 | 1 | 1 | 0 | 1 | 1 | 1 | 0 | 0 | 0 | 1 | 0 | 6 |
| O Aynaud et al. (1998) | 1 | 1 | 1 | 1 | 1 | 0 | 0 | 0 | 0 | 0 | 0 | 5 |
| Q L Lu et al. (1997) | 1 | 1 | 1 | 0 | 1 | 1 | 0 | 1 | 0 | 0 | 0 | 6 |
| K Cooper et al. (1997) | 1 | 1 | 1 | 0 | 1 | 0 | 0 | 0 | 0 | 0 | 0 | 4 |
| P Tenti et al. (1996) | 1 | 1 | 1 | 0 | 1 | 1 | 0 | 0 | 0 | 0 | 0 | 5 |
| A Lopez-Beltran et al. (1996) | 1 | 1 | 0 | 1 | 1 | 0 | 0 | 1 | 0 | 0 | 1 | 6 |
| N R Boucher et al. (1996) | 1 | 1 | 1 | 0 | 1 | 0 | 0 | 0 | 0 | 0 | 0 | 4 |
| K H Kim et al. (1995) | 1 | 1 | 0 | 1 | 1 | 0 | 0 | 0 | 0 | 0 | 0 | 4 |
| D Kamel et al. (1995) | 1 | 1 | 1 | 1 | 1 | 0 | 0 | 0 | 0 | 0 | 0 | 5 |
| V Gopalkrishna et al. (1995) | 1 | 1 | 0 | 1 | 1 | 0 | 0 | 0 | 0 | 0 | 0 | 4 |
| F Chang et al. (1994) | 1 | 1 | 1 | 1 | 1 | 1 | 0 | 0 | 0 | 0 | 0 | 6 |
| G P Mincione et al. (1994) | 1 | 1 | 0 | 0 | 1 | 1 | 0 | 0 | 0 | 0 | 0 | 4 |
| D R Saltzstein et al. (1993) | 1 | 1 | 0 | 0 | 1 | 1 | 0 | 0 | 0 | 0 | 0 | 4 |
| S P Wilczynski et al. (1993) | 1 | 1 | 0 | 1 | 1 | 1 | 0 | 0 | 0 | 0 | 0 | 5 |
| Y F Shibutani et al. (1992) | 1 | 1 | 0 | 1 | 1 | 1 | 0 | 0 | 0 | 0 | 0 | 5 |
| M. A. Knowles et al. (1992) | 1 | 1 | 0 | 1 | 1 | 1 | 0 | 0 | 0 | 0 | 0 | 5 |
| C. Chetsanga et al. (1992) | 1 | 1 | 0 | 1 | 1 | 1 | 0 | 0 | 0 | 0 | 0 | 5 |
| J K Kulski et al. (1990) | 1 | 1 | 0 | 0 | 1 | 1 | 0 | 0 | 0 | 0 | 0 | 4 |

An item would be scored ‘0’ if it was answered ‘NO’ or ‘UNCLEAR’; if it was answered ‘YES’, then the item scored ‘1’.
