# Supplementary material for: The association between human papillomavirus and bladder cancer: Evidence from meta‐analysis and two‐sample mendelian randomization
Source: J Med Virol. 2022 Oct 25;95(1):e28208. doi: 10.1002/jmv.28208 (PMC10092419; doi:10.1002/jmv.28208)
Supplement: Supplementary file 13 — Supporting information. [file JMV-95-0-s011.docx]

**Table S4 Newcastle–Ottawa Scale for assessing the quality of case control studies in meta-analysis.**

| **Study (year)** | **selection** | | | | **Comparability** | **Exposure** | | | **score** |
| --- | --- | --- | --- | --- | --- | --- | --- | --- | --- |
|  | **Is the case definition adequate** | **Representativeness of the cases** | **Selection of Controls** | **Definition of Controls** | **Comparability of cases and controls on the basis of the design or analysis** | **Ascertainment of exposure** | **Same method of ascertainment for cases and controls** | **Non-Response rate** |  |
| Mehmet Sarier et al. 2020 | ★ | ★ |  | ★ | ★ | ★ | ★ | ★ | 7 |
| Sebastian C. Schmid et al. 2015 | ★ | ★ |  | ★ |  | ★ | ★ |  | 5 |
| Sung Han Kim et al. 2014 | ★ | ★ |  | ★ | ★★ | ★ | ★ | ★ | 8 |
| Julie Steinestel et al. 2013 | ★ | ★ |  | ★ | ★ | ★ | ★ | ★ | 7 |
| Kazuyoshi Shigehara et al. 2013 | ★ | ★ |  | ★ |  | ★ | ★ |  | 5 |
| Olfat Gamil Shaker et al. 2013 | ★ | ★ |  | ★ |  | ★ | ★ | ★ | 6 |
| Georgios I. Panagiotakis et al. 2013 | ★ | ★ |  | ★ |  | ★ | ★ | ★ | 6 |
| Noâma Berrada et al. 2013 | ★ | ★ |  | ★ |  | ★ | ★ | ★ | 6 |
| J Polesel et al. 2012 | ★ | ★ |  | ★ | ★★ | ★ | ★ |  | 7 |
| Kazuyoshi Shigehara et al. 2011 | ★ | ★ |  | ★ | ★★ | ★ | ★ |  | 7 |
| Tommaso Cai et al. 2011 | ★ | ★ |  | ★ | ★★ | ★ | ★ |  | 7 |
| H. Yang et al. 2005 | ★ | ★ |  | ★ |  | ★ | ★ |  | 5 |
| Alberto L. Escudero et al. 2005 | ★ | ★ |  | ★ |  | ★ | ★ |  | 5 |
| MR Barghi et al. 2005 | ★ | ★ |  | ★ | ★★ | ★ | ★ |  | 7 |
| D Fioriti et al. 2003 | ★ |  |  | ★ |  | ★ | ★ |  | 4 |
| Zheng Shan et al. 2002 | ★ | ★ | ★ | ★ |  | ★ | ★ | ★ | 7 |
| T Chen et al. 2000 | ★ | ★ |  | ★ |  | ★ | ★ | ★ | 6 |
| M I Tekin et al. 1999 | ★ | ★ |  | ★ |  | ★ | ★ | ★ | 6 |
| K W Chan et al. 1997 | ★ | ★ |  | ★ | ★ | ★ | ★ | ★ | 7 |
| M Ludwig et al. 1996 | ★ | ★ |  | ★ | ★ | ★ | ★ | ★ | 7 |
| Z Smetana et al. 1995 | ★ | ★ |  | ★ | ★ | ★ | ★ | ★ | 7 |
| A M Aglianò et al.1994 | ★ | ★ |  | ★ | ★ | ★ | ★ |  | 6 |
| S T Yu et al.1993 | ★ | ★ |  | ★ |  | ★ | ★ |  | 5 |
| Khurshid Anwar et al.1992 | ★ | ★ |  | ★ | ★★ | ★ | ★ |  | 7 |
| P Bryant et al.1991 | ★ | ★ | ★ | ★ |  | ★ | ★ |  | 6 |

A study can be awarded a maximum of one point for each numbered item within the Selection and Outcome categories and a maximum of two points can be given for Comparability.
