# Supplementary material for: The association between human papillomavirus and bladder cancer: Evidence from meta‐analysis and two‐sample mendelian randomization
Source: J Med Virol. 2022 Oct 25;95(1):e28208. doi: 10.1002/jmv.28208 (PMC10092419; doi:10.1002/jmv.28208)
Supplement: Supplementary file 14 — Supporting information. [file JMV-95-0-s021.docx]

**Table S5. Meta-regression analysis of factors affecting heterogeneity for HPV prevalence.**

| **Variable** | **Coefficient (95%CI)** | **SE** | **Statistical significance (p)** |
| --- | --- | --- | --- |
| **The percentage of male patients** | 0.4070 (-0.2367, 1.0507) | 0.3284 | 0.2153 |
| **Smoking rate** | -0.0687 (-0.8005, 0.6631) | 0.3734 | 0.8540 |

**HPV, human papilloma virus; CI, Confidence Interval; se, SE, standard error**
