# Supplementary material for: The association between human papillomavirus and bladder cancer: Evidence from meta‐analysis and two‐sample mendelian randomization
Source: J Med Virol. 2022 Oct 25;95(1):e28208. doi: 10.1002/jmv.28208 (PMC10092419; doi:10.1002/jmv.28208)
Supplement: Supplementary file 15 — Supporting information. [file JMV-95-0-s013.docx]

**Table S6. Meta-regression analysis of factors affecting heterogeneity for the association between HPV infection and bladder cancer risk.**

| **Variable** | **Coefficient (95%CI)** | **SE** | **Statistical significance (p)** |
| --- | --- | --- | --- |
| **Age** | -0.0274 (-0.1182, 0.0634) | 0.0463 | 0.5547 |
| **The percentage of male patients** | 5.1693 (1.3625, 8.9761) | 1.9423 | 0.0078 |
| **Smoking rate** | 0.4844 (-1.2861, 2.2549) | 0.9033 | 0.5918 |
| **Publication year** | -0.0342 (-0.1030, 0.0346) | 0.0351 | 0.3295 |

**HPV, human papilloma virus; CI, Confidence Interval; se, SE, standard error**
