# Supplementary material for: The association between human papillomavirus and bladder cancer: Evidence from meta‐analysis and two‐sample mendelian randomization
Source: J Med Virol. 2022 Oct 25;95(1):e28208. doi: 10.1002/jmv.28208 (PMC10092419; doi:10.1002/jmv.28208)
Supplement: Supplementary file 16 — Supporting information. [file JMV-95-0-s010.docx]

**Table S7. Meta-regression analysis of factors affecting heterogeneity for the association between HPV infection and bladder cancer prognosis.**

| **Variable** | **Coefficient (95%CI)** | **SE** | **Statistical significance (p)** |
| --- | --- | --- | --- |
| **Follow up duration** | -0.0418 (-0.1382, 0.0545) | 0.0492 | 0.3950 |
| **Publication year** | -0.0070 (-0.0400, 0.0261) | 0.0169 | 0.6786 |
| **Age** | -0.0504 (-0.1718, 0.0710) | 0.0619 | 0.4156 |
| **The percentage of male patients** | 0.0078 (-0.0676, 0.0833) | 0.0385 | 0.8388 |
| **Smoking rate** | 0.0396 (-0.0057, 0.0848) | 0.0231 | 0.0867 |
| **HPV 16 prevalence** | 0.0169 (-0.0349, 0.0687) | 0.0264 | 0.5226 |
| **HPV 18 prevalence** | -0.0355 (-0.0681, -0.0029) | 0.0166 | 0.0326 |

**HPV, human papilloma virus; CI, Confidence Interval; se, SE, standard error**
