# Supplementary material for: The association between human papillomavirus and bladder cancer: Evidence from meta‐analysis and two‐sample mendelian randomization
Source: J Med Virol. 2022 Oct 25;95(1):e28208. doi: 10.1002/jmv.28208 (PMC10092419; doi:10.1002/jmv.28208)
Supplement: Supplementary file 17 — Supporting information. [file JMV-95-0-s003.docx]

|  | **id** | **trait** | **note** | **ncase** | **group_name** | **year** | **consortium** | **author** | **sex** | **population** | **unit** | **nsnp** | **sample_size** | **build** | **ncontrol** | **category** | **subcategory** | **ontology** | **mr** | **priority** | **pmid** | **sd** |
| --- | --- | --- | --- | --- | --- | --- | --- | --- | --- | --- | --- | --- | --- | --- | --- | --- | --- | --- | --- | --- | --- | --- |
| 1 | finn-b-CD2_BENIGN_BLADDER_EXALLC | Benign neoplasm: Bladder (all cancers excluded) | CD2_BENIGN_BLADDER_EXALLC | 109 | public | 2021 | NA | NA | Males and Females | European | NA | 16380337 | NA | HG19/GRCh37 | 180709 | Binary | NA | NA | 1 | 0 | NA | NA |
| 2 | ieu-b-4874 | Bladder cancer | NA | 1279 | public | 2021 | NA | Burrows | Males and Females | European | NA | 9904926 | 373295 | HG19/GRCh37 | 372016 | Disease | Cancer | MONDO:0004986 | 1 | 0 | NA | NA |
| 3 | ukb-d-C67 | Diagnoses - main ICD10: C67 Malignant neoplasm of bladder | NA | 1554 | public | 2018 | NA | Neale lab | Males and Females | European | NA | 10267743 | 361194 | HG19/GRCh37 | 359640 | Binary | NA | NA | 1 | 0 | NA | NA |
| 4 | prot-c-2623_54_4 | HPV E7 Type 16 | name=Protein E7_HPV16; chr=NA; start=NA; end=NA; entrez=1489079; uniprot=P03129 | NA | public | 2019 | NA | Suhre K | Males and Females | European | NA | 501428 | NA | HG19/GRCh37 | NA | Continuous | NA | EFO_0007937 | 1 | 0 | 28240269 | NA |
| 5 | finn-b-C3_BLADDER_EXALLC | Malignant neoplasm of bladder (all cancers excluded) | C3_BLADDER_EXALLC | 1115 | public | 2021 | NA | NA | Males and Females | European | NA | 16380305 | NA | HG19/GRCh37 | 174006 | Binary | NA | NA | 1 | 0 | NA | NA |
| 6 | prot-c-2624_31_2 | HPV E7 Type18 | name=Protein E7_HPV18; chr=NA; start=NA; end=NA; entrez=1489089; uniprot=P06788 | NA | public | 2019 | NA | Suhre K | Males and Females | European | NA | 501428 | NA | HG19/GRCh37 | NA | Continuous | NA | EFO_0007937 | 1 | 0 | 28240269 | NA |
