# Supplementary material for: The association between human papillomavirus and bladder cancer: Evidence from meta‐analysis and two‐sample mendelian randomization
Source: J Med Virol. 2022 Oct 25;95(1):e28208. doi: 10.1002/jmv.28208 (PMC10092419; doi:10.1002/jmv.28208)
Supplement: Supplementary file 18 — Supporting information. [file JMV-95-0-s002.docx]

|  | **chr.exposure** | **samplesize.exposure** | **pval.exposure** | **beta.exposure** | **se.exposure** | **pos.exposure** | **id.exposure** | **SNP** | **effect_allele.exposure** | **other_allele.exposure** | **eaf.exposure** | **exposure** | **mr_keep.exposure** | **pval_origin.exposure** | **data_source.exposure** |
| --- | --- | --- | --- | --- | --- | --- | --- | --- | --- | --- | --- | --- | --- | --- | --- |
| 1 | 1 | 994 | 3.39E-05 | 0.1922 | 0.046147 | 71154117 | prot-c-2623_54_4 | rs1334997 | C | T | NA | \|\| id:prot-c-2623_54_4 | TRUE | reported | igd |
| 2 | 1 | 995 | 1.62E-05 | -0.1957 | 0.045165 | 37479392 | prot-c-2623_54_4 | rs548661 | G | T | NA | \|\| id:prot-c-2623_54_4 | TRUE | reported | igd |
| 3 | 1 | 996 | 5.15E-06 | 0.443 | 0.096641 | 1.94E+08 | prot-c-2623_54_4 | rs6670046 | C | T | NA | \|\| id:prot-c-2623_54_4 | TRUE | reported | igd |
| 4 | 1 | 995 | 2.77E-05 | -0.781 | 0.185467 | 2.43E+08 | prot-c-2623_54_4 | rs2919024 | G | A | NA | \|\| id:prot-c-2623_54_4 | TRUE | reported | igd |
| 5 | 2 | 997 | 1.96E-06 | -0.2523 | 0.052716 | 1.79E+08 | prot-c-2623_54_4 | rs9808377 | G | A | NA | \|\| id:prot-c-2623_54_4 | TRUE | reported | igd |
| 6 | 2 | 996 | 3.55E-05 | 0.2447 | 0.058907 | 60866057 | prot-c-2623_54_4 | rs6719607 | A | G | NA | \|\| id:prot-c-2623_54_4 | TRUE | reported | igd |
| 7 | 2 | 994 | 4.72E-05 | -0.7154 | 0.175043 | 55970893 | prot-c-2623_54_4 | rs17047217 | G | A | NA | \|\| id:prot-c-2623_54_4 | TRUE | reported | igd |
| 8 | 3 | 995 | 6.67E-06 | 0.3036 | 0.06705 | 54264197 | prot-c-2623_54_4 | rs9830760 | C | T | NA | \|\| id:prot-c-2623_54_4 | TRUE | reported | igd |
| 9 | 3 | 996 | 3.69E-05 | -0.2369 | 0.057153 | 1.46E+08 | prot-c-2623_54_4 | rs2864426 | G | C | NA | \|\| id:prot-c-2623_54_4 | TRUE | reported | igd |
| 10 | 5 | 997 | 4.18E-05 | 0.3735 | 0.090743 | 27610789 | prot-c-2623_54_4 | rs7447521 | A | C | NA | \|\| id:prot-c-2623_54_4 | TRUE | reported | igd |
| 11 | 6 | 997 | 9.99E-06 | 0.5598 | 0.126081 | 81380831 | prot-c-2623_54_4 | rs2503718 | G | A | NA | \|\| id:prot-c-2623_54_4 | TRUE | reported | igd |
| 12 | 6 | 997 | 3.59E-05 | 0.2236 | 0.053867 | 65433784 | prot-c-2623_54_4 | rs985915 | A | G | NA | \|\| id:prot-c-2623_54_4 | TRUE | reported | igd |
| 13 | 6 | 997 | 2.14E-05 | 0.2119 | 0.049614 | 24931083 | prot-c-2623_54_4 | rs216259 | A | G | NA | \|\| id:prot-c-2623_54_4 | TRUE | reported | igd |
| 14 | 9 | 993 | 8.34E-06 | -0.2052 | 0.045804 | 1358244 | prot-c-2623_54_4 | rs682927 | T | G | NA | \|\| id:prot-c-2623_54_4 | TRUE | reported | igd |
| 15 | 9 | 997 | 4.49E-06 | 0.2443 | 0.052959 | 95589160 | prot-c-2623_54_4 | rs7850036 | T | C | NA | \|\| id:prot-c-2623_54_4 | TRUE | reported | igd |
| 16 | 11 | 996 | 3.55E-06 | -0.4405 | 0.094467 | 80884316 | prot-c-2623_54_4 | rs10897761 | C | T | NA | \|\| id:prot-c-2623_54_4 | TRUE | reported | igd |
| 17 | 13 | 996 | 1.34E-05 | 0.3406 | 0.077851 | 1.1E+08 | prot-c-2623_54_4 | rs7339361 | C | T | NA | \|\| id:prot-c-2623_54_4 | TRUE | reported | igd |
| 18 | 13 | 997 | 3.60E-05 | 0.3514 | 0.084654 | 50005050 | prot-c-2623_54_4 | rs28692644 | G | A | NA | \|\| id:prot-c-2623_54_4 | TRUE | reported | igd |
| 19 | 13 | 990 | 9.84E-06 | 0.2953 | 0.066449 | 48650492 | prot-c-2623_54_4 | rs41284209 | G | A | NA | \|\| id:prot-c-2623_54_4 | TRUE | reported | igd |
| 20 | 14 | 997 | 3.90E-06 | -0.3348 | 0.072124 | 87365603 | prot-c-2623_54_4 | rs8016986 | T | C | NA | \|\| id:prot-c-2623_54_4 | TRUE | reported | igd |
| 21 | 17 | 997 | 1.56E-05 | -0.2013 | 0.046361 | 9713505 | prot-c-2623_54_4 | rs4791360 | A | G | NA | \|\| id:prot-c-2623_54_4 | TRUE | reported | igd |
| 22 | 18 | 994 | 2.46E-05 | 0.2291 | 0.054059 | 37304602 | prot-c-2623_54_4 | rs1513688 | T | C | NA | \|\| id:prot-c-2623_54_4 | TRUE | reported | igd |
| 23 | 18 | 995 | 2.44E-05 | 0.2107 | 0.049693 | 44040660 | prot-c-2623_54_4 | rs17766830 | C | T | NA | \|\| id:prot-c-2623_54_4 | TRUE | reported | igd |
| 24 | 1 | 993 | 4.90E-05 | 0.3317 | 0.081339 | 75700632 | prot-c-2624_31_2 | rs12743566 | G | A | NA | \|\| id:prot-c-2624_31_2 | TRUE | reported | igd |
| 25 | 1 | 997 | 2.34E-05 | 0.3925 | 0.092353 | 1.69E+08 | prot-c-2624_31_2 | rs35449613 | T | C | NA | \|\| id:prot-c-2624_31_2 | TRUE | reported | igd |
| 26 | 2 | 994 | 1.07E-05 | -0.1953 | 0.044136 | 1.17E+08 | prot-c-2624_31_2 | rs4849449 | T | C | NA | \|\| id:prot-c-2624_31_2 | TRUE | reported | igd |
| 27 | 4 | 990 | 1.32E-05 | 0.3344 | 0.076365 | 58935390 | prot-c-2624_31_2 | rs930937 | T | G | NA | \|\| id:prot-c-2624_31_2 | TRUE | reported | igd |
| 28 | 5 | 986 | 1.00E-05 | -0.1927 | 0.043401 | 79809658 | prot-c-2624_31_2 | rs249011 | T | A | NA | \|\| id:prot-c-2624_31_2 | TRUE | reported | igd |
| 29 | 5 | 996 | 3.31E-05 | -0.1928 | 0.046235 | 1.19E+08 | prot-c-2624_31_2 | rs257969 | C | T | NA | \|\| id:prot-c-2624_31_2 | TRUE | reported | igd |
| 30 | 5 | 994 | 5.86E-06 | -0.2211 | 0.048529 | 6621031 | prot-c-2624_31_2 | rs4702371 | G | A | NA | \|\| id:prot-c-2624_31_2 | TRUE | reported | igd |
| 31 | 8 | 996 | 6.16E-06 | 0.2232 | 0.049098 | 73443576 | prot-c-2624_31_2 | rs4738265 | T | C | NA | \|\| id:prot-c-2624_31_2 | TRUE | reported | igd |
| 32 | 8 | 988 | 1.09E-05 | -0.2385 | 0.053947 | 84495551 | prot-c-2624_31_2 | rs7820513 | A | G | NA | \|\| id:prot-c-2624_31_2 | TRUE | reported | igd |
| 33 | 10 | 991 | 3.12E-05 | 0.5772 | 0.137954 | 59333071 | prot-c-2624_31_2 | rs2393316 | G | A | NA | \|\| id:prot-c-2624_31_2 | TRUE | reported | igd |
| 34 | 11 | 996 | 2.24E-05 | -0.3173 | 0.074501 | 73259543 | prot-c-2624_31_2 | rs11235802 | C | A | NA | \|\| id:prot-c-2624_31_2 | TRUE | reported | igd |
| 35 | 12 | 982 | 2.70E-06 | -0.2042 | 0.043254 | 3818665 | prot-c-2624_31_2 | rs241993 | C | T | NA | \|\| id:prot-c-2624_31_2 | TRUE | reported | igd |
| 36 | 16 | 997 | 4.63E-05 | 0.179 | 0.043744 | 8510914 | prot-c-2624_31_2 | rs3901280 | C | T | NA | \|\| id:prot-c-2624_31_2 | TRUE | reported | igd |
