# Supplementary material for: The association between human papillomavirus and bladder cancer: Evidence from meta‐analysis and two‐sample mendelian randomization
Source: J Med Virol. 2022 Oct 25;95(1):e28208. doi: 10.1002/jmv.28208 (PMC10092419; doi:10.1002/jmv.28208)
Supplement: Supplementary file 19 — Supporting information. [file JMV-95-0-s016.docx]

|  | **id_exposure** | **id_outcome** | **outcome** | **exposure** | **egger_intercept** | **se** | **p_value** |
| --- | --- | --- | --- | --- | --- | --- | --- |
| 1 | prot-c-2623_54_4 | finn-b-C3_BLADDER_EXALLC | Malignant neoplasm of bladder (all cancers excluded) \|\| id:finn-b-C3_BLADDER_EXALLC | \|\| id:prot-c-2623_54_4 | -0.01779 | 0.038774 | 0.651312 |
| 2 | prot-c-2623_54_4 | finn-b-CD2_BENIGN_BLADDER_EXALLC | Benign neoplasm: Bladder (all cancers excluded) \|\| id:finn-b-CD2_BENIGN_BLADDER_EXALLC | \|\| id:prot-c-2623_54_4 | -0.18912 | 0.129813 | 0.160665 |
| 3 | prot-c-2623_54_4 | ieu-b-4874 | Bladder cancer \|\| id:ieu-b-4874 | \|\| id:prot-c-2623_54_4 | 3.96E-05 | 0.000121 | 0.747342 |
| 4 | prot-c-2623_54_4 | ukb-d-C67 | Diagnoses - main ICD10: C67 Malignant neoplasm of bladder \|\| id:ukb-d-C67 | \|\| id:prot-c-2623_54_4 | -9.32E-05 | 0.000157 | 0.55954 |
| 5 | prot-c-2624_31_2 | finn-b-C3_BLADDER_EXALLC | Malignant neoplasm of bladder (all cancers excluded) \|\| id:finn-b-C3_BLADDER_EXALLC | \|\| id:prot-c-2624_31_2 | -0.04637 | 0.05527 | 0.42106 |
| 6 | prot-c-2624_31_2 | finn-b-CD2_BENIGN_BLADDER_EXALLC | Benign neoplasm: Bladder (all cancers excluded) \|\| id:finn-b-CD2_BENIGN_BLADDER_EXALLC | \|\| id:prot-c-2624_31_2 | -0.09505 | 0.168393 | 0.584884 |
| 7 | prot-c-2624_31_2 | ieu-b-4874 | Bladder cancer \|\| id:ieu-b-4874 | \|\| id:prot-c-2624_31_2 | 6.41E-05 | 0.000217 | 0.774643 |
| 8 | prot-c-2624_31_2 | ukb-d-C67 | Diagnoses - main ICD10: C67 Malignant neoplasm of bladder \|\| id:ukb-d-C67 | \|\| id:prot-c-2624_31_2 | -0.00042 | 0.000229 | 0.099267 |
