# Supplementary material for: The association between human papillomavirus and bladder cancer: Evidence from meta‐analysis and two‐sample mendelian randomization
Source: J Med Virol. 2022 Oct 25;95(1):e28208. doi: 10.1002/jmv.28208 (PMC10092419; doi:10.1002/jmv.28208)
Supplement: Supplementary file 20 — Supporting information. [file JMV-95-0-s001.docx]

|  | **id_exposure** | **id_outcome** | **outcome** | **exposure** | **method** | **Q** | **Q_df** | **Q_p_value** |
| --- | --- | --- | --- | --- | --- | --- | --- | --- |
| 1 | prot-c-2623_54_4 | finn-b-C3_BLADDER_EXALLC | Malignant neoplasm of bladder (all cancers excluded) \|\| id:finn-b-C3_BLADDER_EXALLC | \|\| id:prot-c-2623_54_4 | MR Egger | 14.11926 | 20 | 0.824398 |
| 2 | prot-c-2623_54_4 | finn-b-C3_BLADDER_EXALLC | Malignant neoplasm of bladder (all cancers excluded) \|\| id:finn-b-C3_BLADDER_EXALLC | \|\| id:prot-c-2623_54_4 | Inverse variance weighted | 14.32977 | 21 | 0.855022 |
| 3 | prot-c-2623_54_4 | finn-b-CD2_BENIGN_BLADDER_EXALLC | Benign neoplasm: Bladder (all cancers excluded) \|\| id:finn-b-CD2_BENIGN_BLADDER_EXALLC | \|\| id:prot-c-2623_54_4 | MR Egger | 23.94544 | 20 | 0.244783 |
| 4 | prot-c-2623_54_4 | finn-b-CD2_BENIGN_BLADDER_EXALLC | Benign neoplasm: Bladder (all cancers excluded) \|\| id:finn-b-CD2_BENIGN_BLADDER_EXALLC | \|\| id:prot-c-2623_54_4 | Inverse variance weighted | 26.48673 | 21 | 0.188495 |
| 5 | prot-c-2623_54_4 | ieu-b-4874 | Bladder cancer \|\| id:ieu-b-4874 | \|\| id:prot-c-2623_54_4 | MR Egger | 17.57504 | 19 | 0.55097 |
| 6 | prot-c-2623_54_4 | ieu-b-4874 | Bladder cancer \|\| id:ieu-b-4874 | \|\| id:prot-c-2623_54_4 | Inverse variance weighted | 17.68189 | 20 | 0.608355 |
| 7 | prot-c-2623_54_4 | ukb-d-C67 | Diagnoses - main ICD10: C67 Malignant neoplasm of bladder \|\| id:ukb-d-C67 | \|\| id:prot-c-2623_54_4 | MR Egger | 22.4469 | 20 | 0.316761 |
| 8 | prot-c-2623_54_4 | ukb-d-C67 | Diagnoses - main ICD10: C67 Malignant neoplasm of bladder \|\| id:ukb-d-C67 | \|\| id:prot-c-2623_54_4 | Inverse variance weighted | 22.84216 | 21 | 0.352452 |
| 9 | prot-c-2624_31_2 | finn-b-C3_BLADDER_EXALLC | Malignant neoplasm of bladder (all cancers excluded) \|\| id:finn-b-C3_BLADDER_EXALLC | \|\| id:prot-c-2624_31_2 | MR Egger | 3.890127 | 10 | 0.952167 |
| 10 | prot-c-2624_31_2 | finn-b-C3_BLADDER_EXALLC | Malignant neoplasm of bladder (all cancers excluded) \|\| id:finn-b-C3_BLADDER_EXALLC | \|\| id:prot-c-2624_31_2 | Inverse variance weighted | 4.594088 | 11 | 0.949222 |
| 11 | prot-c-2624_31_2 | finn-b-CD2_BENIGN_BLADDER_EXALLC | Benign neoplasm: Bladder (all cancers excluded) \|\| id:finn-b-CD2_BENIGN_BLADDER_EXALLC | \|\| id:prot-c-2624_31_2 | MR Egger | 5.016031 | 10 | 0.890105 |
| 12 | prot-c-2624_31_2 | finn-b-CD2_BENIGN_BLADDER_EXALLC | Benign neoplasm: Bladder (all cancers excluded) \|\| id:finn-b-CD2_BENIGN_BLADDER_EXALLC | \|\| id:prot-c-2624_31_2 | Inverse variance weighted | 5.334638 | 11 | 0.913884 |
| 13 | prot-c-2624_31_2 | ieu-b-4874 | Bladder cancer \|\| id:ieu-b-4874 | \|\| id:prot-c-2624_31_2 | MR Egger | 5.896673 | 8 | 0.658805 |
| 14 | prot-c-2624_31_2 | ieu-b-4874 | Bladder cancer \|\| id:ieu-b-4874 | \|\| id:prot-c-2624_31_2 | Inverse variance weighted | 5.984389 | 9 | 0.74148 |
| 15 | prot-c-2624_31_2 | ukb-d-C67 | Diagnoses - main ICD10: C67 Malignant neoplasm of bladder \|\| id:ukb-d-C67 | \|\| id:prot-c-2624_31_2 | MR Egger | 9.492199 | 10 | 0.486114 |
| 16 | prot-c-2624_31_2 | ukb-d-C67 | Diagnoses - main ICD10: C67 Malignant neoplasm of bladder \|\| id:ukb-d-C67 | \|\| id:prot-c-2624_31_2 | Inverse variance weighted | 12.79359 | 11 | 0.30703 |
