# Supplementary material for: The association between human papillomavirus and bladder cancer: Evidence from meta‐analysis and two‐sample mendelian randomization
Source: J Med Virol. 2022 Oct 25;95(1):e28208. doi: 10.1002/jmv.28208 (PMC10092419; doi:10.1002/jmv.28208)
Supplement: Supplementary file 21 — Supporting information. [file JMV-95-0-s018.docx]

|  | **id.exposure** | **id.outcome** | **outcome** | **exposure** | **method** | **nsnp** | **b** | **se** | **pval** | **lo_ci** | **up_ci** | **or** | **or_lci95** | **or_uci95** |
| --- | --- | --- | --- | --- | --- | --- | --- | --- | --- | --- | --- | --- | --- | --- |
| 1 | prot-c-2623_54_4 | ieu-b-4874 | Bladder cancer \|\| id:ieu-b-4874 | \|\| id:prot-c-2623_54_4 | MR Egger | 21 | 0.000241 | 0.000442 | 0.591421 | -0.00063 | 0.001108 | 1.000241 | 0.999375 | 1.001109 |
| 2 | prot-c-2623_54_4 | ieu-b-4874 | Bladder cancer \|\| id:ieu-b-4874 | \|\| id:prot-c-2623_54_4 | Weighted median | 21 | 0.000329 | 0.000214 | 0.125531 | -9.18E-05 | 0.000749 | 1.000329 | 0.999908 | 1.000749 |
| 3 | prot-c-2623_54_4 | ieu-b-4874 | Bladder cancer \|\| id:ieu-b-4874 | \|\| id:prot-c-2623_54_4 | Inverse variance weighted | 21 | 0.000377 | 0.000153 | 0.013829 | 7.68E-05 | 0.000677 | 1.000377 | 1.000077 | 1.000677 |
| 4 | prot-c-2623_54_4 | ieu-b-4874 | Bladder cancer \|\| id:ieu-b-4874 | \|\| id:prot-c-2623_54_4 | Simple mode | 21 | 0.000417 | 0.000414 | 0.325387 | -0.00039 | 0.001228 | 1.000417 | 0.999606 | 1.001229 |
| 5 | prot-c-2623_54_4 | ieu-b-4874 | Bladder cancer \|\| id:ieu-b-4874 | \|\| id:prot-c-2623_54_4 | Weighted mode | 21 | 0.000347 | 0.000373 | 0.363237 | -0.00038 | 0.001079 | 1.000347 | 0.999616 | 1.00108 |
| 6 | prot-c-2624_31_2 | ieu-b-4874 | Bladder cancer \|\| id:ieu-b-4874 | \|\| id:prot-c-2624_31_2 | MR Egger | 10 | -0.00045 | 0.000954 | 0.649003 | -0.00232 | 0.001419 | 0.999549 | 0.997681 | 1.00142 |
| 7 | prot-c-2624_31_2 | ieu-b-4874 | Bladder cancer \|\| id:ieu-b-4874 | \|\| id:prot-c-2624_31_2 | Weighted median | 10 | -7.62E-05 | 0.0003 | 0.799474 | -0.00066 | 0.000512 | 0.999924 | 0.999336 | 1.000512 |
| 8 | prot-c-2624_31_2 | ieu-b-4874 | Bladder cancer \|\| id:ieu-b-4874 | \|\| id:prot-c-2624_31_2 | Inverse variance weighted | 10 | -0.00018 | 0.000228 | 0.43869 | -0.00062 | 0.000271 | 0.999823 | 0.999376 | 1.000271 |
| 9 | prot-c-2624_31_2 | ieu-b-4874 | Bladder cancer \|\| id:ieu-b-4874 | \|\| id:prot-c-2624_31_2 | Simple mode | 10 | -0.00014 | 0.00047 | 0.768466 | -0.00106 | 0.000779 | 0.999857 | 0.998936 | 1.000779 |
| 10 | prot-c-2624_31_2 | ieu-b-4874 | Bladder cancer \|\| id:ieu-b-4874 | \|\| id:prot-c-2624_31_2 | Weighted mode | 10 | -0.00013 | 0.000462 | 0.783757 | -0.00104 | 0.000774 | 0.999869 | 0.998965 | 1.000775 |
