# Supplementary material for: The association between human papillomavirus and bladder cancer: Evidence from meta‐analysis and two‐sample mendelian randomization
Source: J Med Virol. 2022 Oct 25;95(1):e28208. doi: 10.1002/jmv.28208 (PMC10092419; doi:10.1002/jmv.28208)
Supplement: Supplementary file 22 — Supporting information. [file JMV-95-0-s008.docx]

**Figure S1: Subgroup analyses for high-risk HPV prevalence in bladder cancer cases stratified by continent, histological type and study type.**

Note: CI, confidence interval; No., number; TCC, transitional cell carcinoma; SCC, squamous cell carcinoma.

**Figure S2: Publication bias for the included studies on HPV prevalence.**

**A.** Funnel plot. **B.** Galbraith plot. Effect sizes as z-scores plotted as a function of the inverse standard error for each study reported in this study. The middle line is the line of best fit, while upper and lower dashed lines represent upper and lower 95% confidence limits. **C.** Trim and fill funnel plot.

**Figure S3: Trim and fill method and sensitivity analysis.**

**A.** Filled forest plot. **B.** Cumulative meta-analysis by stepwise addition of the included studies. **C.** Sensitivity analysis by stepwise omission of the included studies.

**Figure S4: Meta-regression for HPV prevalence and covariates. A.** Percentage of male patients. **B.** Smoking rate. Each dot represents an individual study. Symbol size represents sample size.

**Figure S5: Trim and fill method and detection of publication bias for the included studies on OR of BCa.**

**A.** Funnel plot. **B.** Trim and fill funnel plot. **C.** Filled forest plot. **D.** Galbraith plot. **E.** The L’Abbe plot for incidences of BCa. Each dot represents an individual study. Symbol size represents sample size.

**Figure S6: Sensitivity analysis and Cumulative meta-analysis for included studies on OR of BCa.**

**A.** Cumulative meta-analysis by stepwise adding the included studies. **B.** Sensitivity analysis by stepwise omitting the included studies.

**Figure S7:** **Meta-regression for OR of BCa and covariates. A.** Percentage of male patients. **B.** Publication year. **C.** Age. **D.** Smoking rate. Each dot represents an individual study. Symbol size represents the sample size.

**Figure S8: Sensitivity analysis and detection of publication bias for the included studies on RR of BCa progression.**

**A.** Funnel plot. **B.** Trim and fill funnel plot. **C.** Filled forest plot. **D.** Cumulative meta-analysis by stepwise addition of included studies. **E.** Sensitivity analysis by stepwise omission of the included studies. **F.** Galbraith plot. **G.** L’Abbe plot for RR of BCa progression.

**Figure S9: Meta-regression for RR of BCa progression and covariates.** **A.** Duration of follow up. **B.** Smoking rate. **C.** Percentage of male patients. **D.** Age. **E.** Publication year. **F.** Prevalence of HPV 16. **G.** Prevalence of HPV 18. Each dot represents an individual study. Symbol size represents sample size.

**Table 1: Characteristics of included studies in the systematic review and meta-analysis.**

**Table S1: Details of Search Strategy to Retrieve the Studies using PubMed (Medline), Embase, Cochrane and Web of Science.**

**Table S2: The prevalence of HPV types in bladder cancer cases of the included studies in this systematic review and meta-analysis.**

**Table S3: Agency for Healthcare Research and Quality (AHRQ) evaluation criteria for assessing the quality of included cross-sectional studies.**

**Table S4: Newcastle–Ottawa Scale for assessing the quality of case control studies in meta-analysis.**

**Table S5: Meta-regression analysis of factors affecting heterogeneity for HPV prevalence.**

**Table S6: Meta-regression analysis of factors affecting heterogeneity for the association between HPV infection and bladder cancer risk.**

**Table S7: Meta-regression analysis of factors affecting heterogeneity for the association between HPV infection and bladder cancer prognosis.**

**Table S8: The data source for two-sample mendelian randomization study.**

**Table S9: 36 single-nucleotide polymorphisms (SNPs) from 4 genome-wide association studies (GWAS) used as instrumental variables to evaluate the causality of HPV E7 protein.**

**Table S10: The pleiotropic effects tested by pleiotropy test.**

**Table S11: Heterogeneity tested by Cochrane Q value.**

**Table S12: Detailed information on SNPs for HPV E7 protein and bladder cancer.**
